# Fibroblast-derived EGF ligand neuregulin 1 induces fetal-like reprogramming of the intestinal epithelium without supporting tumorigenic growth

**DOI:** 10.1242/dmm.049692

**Published:** 2023-04-03

**Authors:** Toni T. Lemmetyinen, Emma W. Viitala, Linnea Wartiovaara, Tuomas Kaprio, Jaana Hagström, Caj Haglund, Pekka Katajisto, Timothy C. Wang, Eva Domènech-Moreno, Saara Ollila

**Affiliations:** ^1^ Translational Cancer Medicine Program, University of Helsinki, 00014 Helsinki, Finland; ^2^ Department of Surgery, University of Helsinki and Helsinki University Hospital, 00290 Helsinki, Finland; ^3^ Department of Pathology, University of Helsinki and Helsinki University Hospital, 00290 Helsinki, Finland; ^4^ Department of Oral Pathology and Radiology, University of Turku, 20014 Turku, Finland; ^5^ Institute of Biotechnology, HiLIFE, University of Helsinki, 00014 Helsinki, Finland; ^6^ Department of Cell and Molecular Biology, Karolinska Institutet, 17177 Stockholm, Sweden; ^7^ Faculty of Biological and Environmental Sciences, University of Helsinki, 00014 Helsinki, Finland; ^8^ Division of Digestive and Liver Diseases, Department of Medicine, Irving Cancer Research Center, Columbia University Medical Center, New York, NY 10032, USA; ^9^ HiLIFE-Helsinki Institute of Life Science, University of Helsinki, 00014 Helsinki, Finland

**Keywords:** Colorectal cancer, EGF, EREG, Intestinal regeneration, NRG1

## Abstract

Growth factors secreted by stromal fibroblasts regulate the intestinal epithelium. Stroma-derived epidermal growth factor (EGF) family ligands are implicated in epithelial regeneration and tumorigenesis, but their specific contributions and associated mechanisms remain unclear. Here, we use primary intestinal organoids modeling homeostatic, injured and tumorigenic epithelia to assess how the fibroblast-derived EGF family ligands neuregulin 1 (NRG1) and epiregulin (EREG) regulate the intestinal epithelium. NRG1 was expressed exclusively in the stroma, robustly increased crypt budding and protected intestinal epithelial organoids from radiation-induced damage. NRG1 also induced regenerative features in the epithelium, including a fetal-like transcriptome, suppression of the Lgr5^+^ stem cell pool and remodeling of the epithelial actin cytoskeleton. Intriguingly, unlike EGF and EREG, NRG1 failed to support the growth of pre-tumorigenic intestinal organoids lacking the tumor suppressor *Apc*, commonly mutated in human colorectal cancer (CRC). Interestingly, high expression of stromal NRG1 was associated with improved survival in CRC cohorts, suggesting a tumor-suppressive function. Our results highlight the power of stromal NRG1 in transcriptional reprogramming and protection of the intestinal epithelium from radiation injury without promoting tumorigenesis.

## Teaser

**Summary:** Pathways involved in regenerative responses may also promote tumorigenesis; however, the fibroblast-derived EGF ligand neuregulin 1 protects the intestinal epithelium from injury, but does not support tumorigenic growth.

## INTRODUCTION

The gastrointestinal (GI) epithelium is formed by a single layer of constantly renewing cells structured into invaginations called crypts in the colon and alternating crypt and villus units in the small intestine. The renewal is fueled by the proliferative, Lgr5-expressing stem cells situated in the bottom of the crypts, and differentiation occurs while the cells migrate towards the gut lumen. In homeostatic conditions, growth factor gradients generated by spatially restricted subtypes of stromal fibroblasts regulate the stemness and maturation of the GI epithelium: for example, fibroblasts close to the stem cell zone secrete stemness-promoting factors such as the Wnt agonists R-spondins (RSPOs) and the BMP inhibitor gremlin 1 (GREM1), whereas fibroblasts higher in the crypt secrete BMP ligands and promote differentiation (Aoki et al., 2016; Greicius et al., 2018; Jacob et al., 2022; McCarthy et al., 2020a). Lack of Wnt ligand secretion from subepithelial fibroblasts leads to epithelial defects, highlighting the importance of the mesenchymal niche in maintenance of the GI epithelium (Degirmenci et al., 2018; Shoshkes-Carmel et al., 2018).

The thin structure of the GI epithelium makes it efficient for nutrient transport but prone to injury. Damage to the epithelium induced, for example, by a local wound, γ-irradiation or chemically induced inflammation leads to loss of the epithelial barrier, followed by gradual restoration of the homeostasis through a hypertrophic regenerative state involving coverage of the wound bed with immature epithelial cells, which is then followed by new crypt formation through crypt fission, finally resulting in a restored epithelium with mature crypt organization (Miyoshi et al., 2012). Interestingly, the regeneration process has recently been shown to be associated with a transient reprogramming of the epithelium into a fetal-like state, characterized by increased expression of, for example, Ly6 and annexin family genes, activation of YAP signaling, suppression of WNT signaling, as well as temporary stem cell quiescence (Cheung et al., 2020; Gregorieff et al., 2015; Ohara et al., 2022; Yui et al., 2018). In addition, extensive remodeling of the actin cytoskeleton, including changes in the apical filamentous actin (F-actin) belt, occurs during regeneration (Ohara et al., 2022; Ubelmann et al., 2013; Yui et al., 2018). Upon GI damage, fibroblasts alter their transcriptome while retaining topology, thus representing potential sources of the regenerative signals (Melissari et al., 2022). Indeed, fibroblast-derived prostaglandin E2 (PGE2) has been shown to induce fetal-like changes in the intestinal epithelium and to promote regeneration (Roulis et al., 2020). Also, smooth muscle-specific MMP17 was shown to be involved in the regenerative reprogramming (Martín-Alonso et al., 2021). However, the molecular mechanisms coordinating the repair are not fully understood.

Epidermal growth factor (EGF) represents an important growth factor for GI epithelium. During intestinal homeostasis, EGF derived from Paneth cells supports the proliferation of the adjacent intestinal stem cells (ISCs) (Sato et al., 2011). The normal development of intestinal epithelium in the absence of Paneth cells (Durand et al., 2012) and the fact that exogenous EGF is required for the intestinal epithelial organoid growth *ex vivo* (Sato et al., 2009) suggest a role for non-epithelial sources of EGF ligands. The EGF family consists of 11 related ligands that signal via four receptors: EGFR, ERBB2, ERBB3 and ERBB4, which homodimerize and heterodimerize, and activate intracellular signaling pathways in response to ligand binding. In addition to epithelial EGF, mesenchymal EGF family ligands are active regulators of the intestinal epithelium. In the fruit fly intestine, the EGF ligand *vein* is secreted from the visceral muscle and involved in intestinal regeneration (Jiang et al., 2011), and mesenchyme-derived neuregulin 1 (NRG1) was shown to promote epithelial proliferation during homeostasis and regeneration in mice (Jardé et al., 2020). Interestingly, *NRG1* has also been shown to be expressed in the fetal human intestinal mesenchyme, where it appears to promote intestinal epithelial maturation (Yu et al., 2021) and cellular differentiation (Holloway et al., 2021). Also, epiregulin (EREG) from stromal cells has been proposed to be functionally important in intestinal regeneration (Gregorieff et al., 2015).

Regeneration-promoting stromal signals enhance the growth of the epithelium and thus potentially also promote tumorigenic growth. Indeed, fibroblast-derived PGE2 was shown to promote intestinal tumorigenesis in mice (Roulis et al., 2020). Of the EGF ligands, fibroblast-derived EREG promoted inflammation-associated colonic tumorigenesis (Neufert et al., 2013). In addition, *Ereg* knockout mice were more sensitive to dextran sodium sulphate-induced colonic inflammation, but EREG loss did not affect *Apc* mutation-induced tumorigenesis (Lee et al., 2004). Recently, stromal NRG1, EREG and betacellulin (BTC) were suggested to be regulated by colonic epithelium-derived Indian hedgehog signaling and to be functionally involved in *Apc* mutation-induced tumorigenesis (Westendorp et al., 2021). Demonstrating the complexity of the EGF ligand signaling and location, expression of EREG has been shown to be induced during regeneration in the GI epithelium, and EREG as well as another EGF ligand, amphiregulin (AREG), have been proposed to function as effectors of the epithelial regenerative YAP signaling (Gregorieff et al., 2015; Zhang et al., 2009). Although previous data has indicated that mesenchymal NRG1 shifts the intestinal epithelial cells towards a more undifferentiated identity (Jardé et al., 2020), it is unclear whether fibroblast-derived EGF family growth factors are involved in the regenerative reprogramming of the epithelium into a fetal-like state. Furthermore, it is unclear whether the factors stimulating the regenerative responses could selectively promote the growth of normal/regenerative, but not tumorigenic, epithelia.

Here, we identified NRG1 and EREG as the main fibroblast-derived EGF ligands. By combining single-cell RNA sequencing analysis, a variety of primary fibroblast and primary intestinal epithelial organoid models, mouse intestinal tissue analysis, transcriptomic studies, and colorectal cancer (CRC) patient cohort survival analyses, we show that NRG1, but not EREG, is sufficient to reprogram the intestinal epithelium into a regenerative, fetal-like state without promoting tumorigenic growth.

## RESULTS

### Identification of GI fibroblast-derived EGF family ligands

**Fig. 1.**
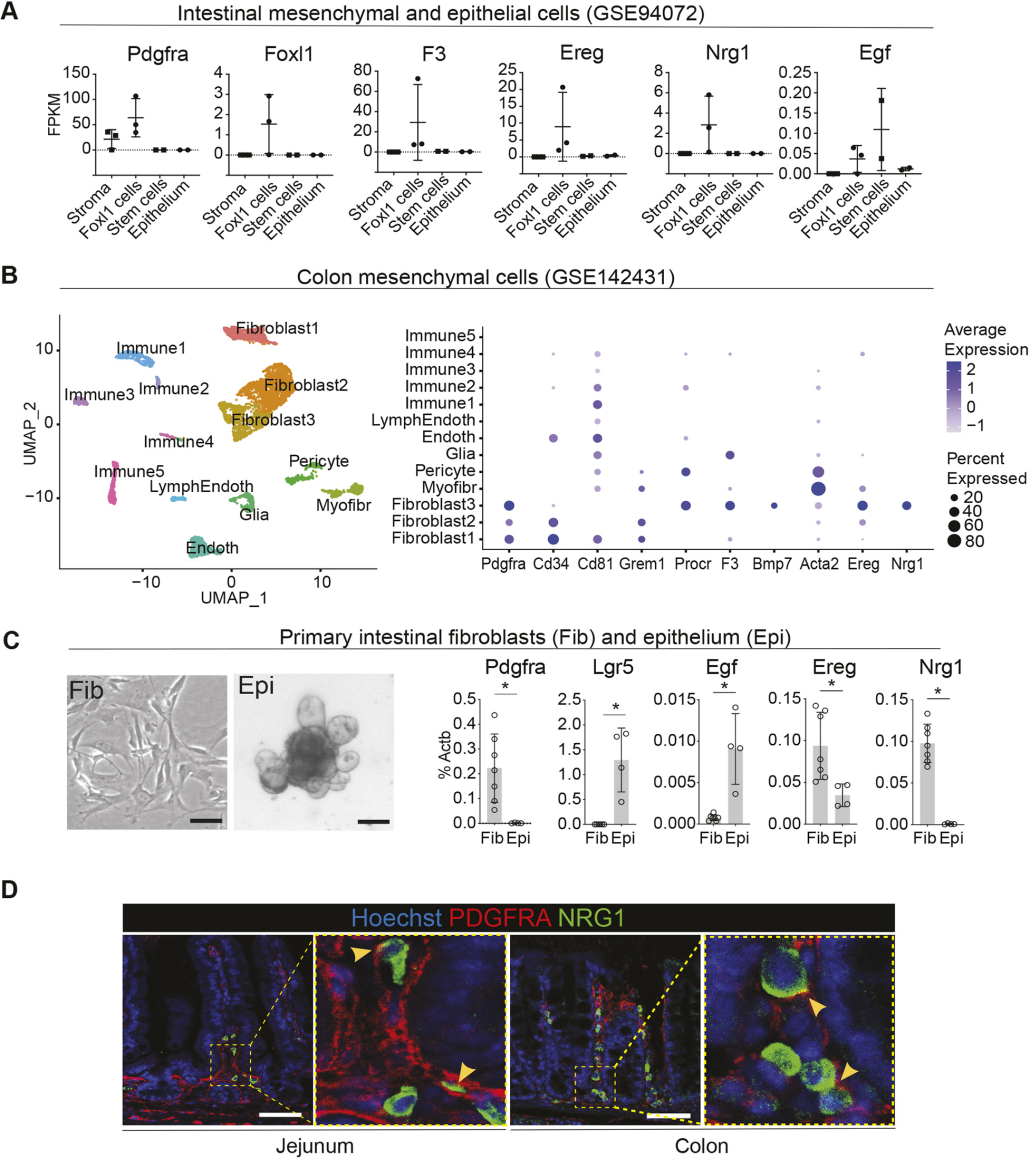
**EGF family ligands EREG and NRG1 are expressed in mouse adult GI fibroblasts.** (A) Comparison of transcript levels of the indicated genes in different cell types of the mouse small intestine. Non-*Foxl1* lineage stromal cells (Stroma), sorted *Foxl1*-lineage cells representing subepithelial fibroblasts (Foxl1 cells), intestinal epithelium stem cells expressing Lgr5 (Stem cells) and non-stem-cell epithelial cells (Epithelium) identified by Shoshkes-Carmel et al. (2018) (GSE94072). FPKM, fragments per kilobase of transcript per million mapped reads. See also Fig. S1A-C. (B) Uniform manifold approximation and projection (UMAP) plot (left) of mouse colon mesenchymal cell clusters identified from Roulis et al. (2020) (GSE142431) and dot plot (right) depicting known markers for fibroblast subpopulations as well as *Ereg* and *Nrg1*. Endoth, endothelial cells; LymphEndoth, lymphatic endothelial cells; Myofibr, myofibroblasts. See also Fig. S1E. (C) Representative images of primary small intestinal fibroblasts (Fib) and small intestinal epithelial organoids (Epi) (left) and mRNA expression levels of the indicated genes (right). Primary intestinal fibroblasts, *n*=7 independent mice; intestinal epithelial organoids, *n*=4 independent mice. Bar graphs depict the mean, error bars represent s.d. Asterisks indicate statistical significance (*P<0.05, two-tailed unpaired *t*-test). Scale bars: 100 µm. (D) Immunofluorescence staining of PDGFRA and NRG1 in homeostatic mouse jejunum and distal colon. Yellow arrowheads mark examples of cells with expression of PDGFRA in the membrane and NRG1 in the cytoplasm. Scale bars: 50 µm.

Subepithelial fibroblasts, marked by *Foxl1* or *Gli1* expression, provide critical growth factors supporting the GI epithelium (Aoki et al., 2016; Degirmenci et al., 2018; Shoshkes-Carmel et al., 2018). To identify which EGF family ligands are expressed in these cells, we investigated the previously published bulk RNA-sequencing dataset including *Foxl1* lineage stromal cells representing subepithelial fibroblasts, non-*Foxl1-*expressing stromal cells, Lgr5-expressing ISCs and non-stem-cell epithelial cells (Shoshkes-Carmel et al., 2018). As expected, the pan-fibroblast marker *Pdgfra* as well as the subepithelial fibroblast markers *Foxl1* and *F3* (Kinchen et al., 2018) were expressed in the *Foxl1* lineage, and *Lgr5* and alkaline phosphatase (*Alpi*) expression confirmed the identity of the Lgr5^+^ ISCs and non-stem-cell epithelium, respectively (Fig. 1A; Fig. S1A). The most highly and specifically expressed EGF family ligands in the subepithelial fibroblasts were *Nrg1* and *Ereg*, whereas *Egf* expression was low (Fig. 1A). Other ligands were not specifically expressed in the stromal fractions but showed either very low expression overall or exhibited epithelial expression to varying degrees (Fig. S1B). This analysis pointed towards *Ereg* and *Nrg1* as the most highly and specifically expressed EGF family ligands in intestinal subepithelial fibroblasts. Of the EGF family receptors, *Egfr* was expressed mostly in Foxl1^+^ stromal cells and Lgr5^+^ ISCs, whereas *Erbb2* and *Erbb3* expression was highest in the non-stem-cell epithelium. No reads for *Erbb4* were found in this dataset (Fig. S1C). Another dataset comparing the transcriptomic profile of Lgr5^+^ ISCs and Lgr5^−^ non-stem-cell epithelial cells (Yan et al., 2017) confirmed the significantly higher expression of *Egfr* in Lgr5^+^ ISCs and *Erbb3* in the Lgr5^−^ non-stem-cell epithelium (Fig. S1D).

Next, we re-analyzed the single-cell RNA sequencing (scRNA-seq) dataset containing data from murine colon mesenchymal cells including fibroblasts, endothelial, immune and glial cells (Roulis et al., 2020) (Fig. 1B). In our analysis, 13 clusters of stromal cells were identified, of which three expressed *Pdgfra* and were classified as fibroblasts. *Pdgfra* levels were highest in the ‘Fibroblast 3’ cluster, which was also positive for *Procr*, *F3 and Bmp7*, markers previously identified in the subepithelial *Pdgfra^high^* cells (‘telocytes’), whereas the ‘Fibroblast 1’ and ‘Fibroblast 2’ clusters expressed *Cd34* and *Grem1*, indicating similarities with the previously described crypt base fibroblasts (Brügger et al., 2020; McCarthy et al., 2020a; Melissari et al., 2022; Stzepourginski et al., 2017). The ‘Fibroblast 1’ cluster also expressed *Cd81*, in line with the previously described trophocytes (McCarthy et al., 2020b). Interestingly, both *Ereg* and *Nrg1* were predominantly expressed in the ‘Fibroblast 3’ population (Fig. 1B). This was consistent with the reported expression of *NRG1* in the *F3*^+^ subepithelial fibroblasts of the human fetal intestine (Holloway et al., 2021; Yu et al., 2021) and suggests that in adult mice, *Ereg* and *Nrg1* are primarily expressed in *Pdgfra^high^* fibroblasts. Of the other EGF family ligands, *Hbegf* was expressed in the *Pecam1*-expressing endothelium and *S100b*-expressing glial cells, and low expression of *Areg* was observed in the ‘Fibroblast 3’ cluster and in some of the Cd45^+^ immune clusters. No or very low expression of other EGF family ligands was noted in this dataset. *Egfr* expression was highest in the ‘Fibroblast 1’ cluster, whereas *Erbb3* expression was noted in glia (Fig. S1E).

To verify the *in silico* results, we profiled the gene expression of *Ereg* and *Nrg1* in primary mouse intestinal fibroblasts and intestinal epithelium using quantitative real-time PCR (qRT-PCR). Fibroblast-specific expression of *Pdgfra* and epithelium-specific expression of *Lgr5* verified the purity of the cell populations (Fig. 1C). Indeed, we found that *Nrg1* expression was exclusively restricted to fibroblasts, whereas *Ereg* was enriched in the fibroblasts but also present at lower levels in the epithelium. In comparison, *Egf* was expressed at low levels mainly in the epithelium (Fig. 1C). Similarly, *in situ* hybridization using RNAscope showed *Ereg* expression both in the stroma and in the epithelium, whereas *Nrg1* expression was restricted to stromal areas (Fig. S2A). Immunofluorescence staining confirmed the exclusively stromal expression of NRG1 in the intestine and colon, which in the intestine was most prevalent in the crypt-villus junction and lower villi (Fig. 1D). Interestingly, the expression of *NRG1* in the human fetal intestine was shown to localize to the crypt bottom (Holloway et al., 2021), indicating either developmental stage- or species-dependent differences in the expression site. The expression of NRG1 was cytoplasmic, whereas PDGFRA localized to cell membranes (Fig. 1D). In conclusion, the *in silico* analysis, verified by qRT-PCR in primary intestinal fibroblasts, demonstrated that during homeostasis, *Nrg1* and *Ereg* represent the main GI fibroblast-derived EGF ligands, of which *Nrg1* expression is exclusively stromal.

### *Ereg* and *Nrg1* expression is increased during GI regeneration

**Fig. 2.**
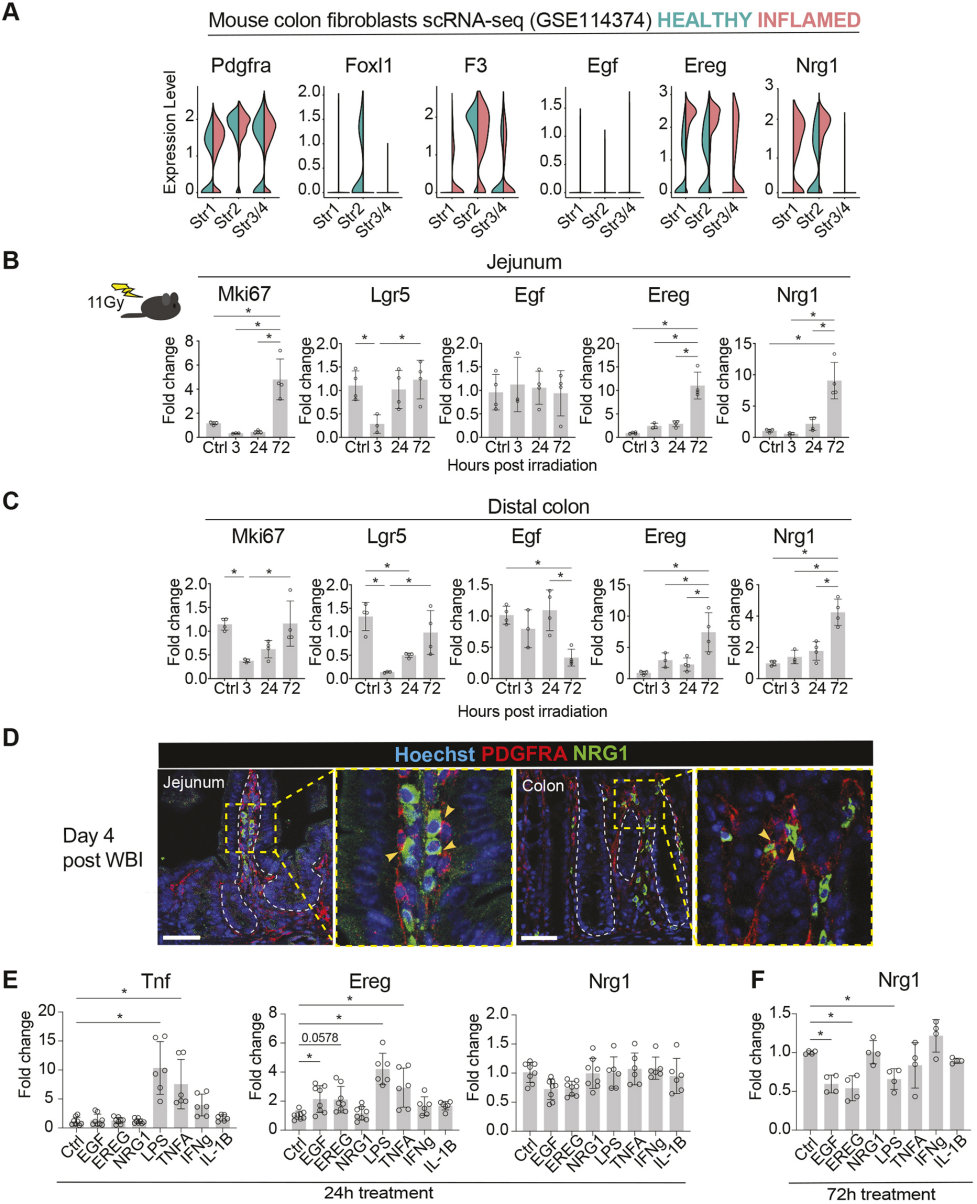
***Ereg* and *Nrg1* gene expression is induced during intestinal regeneration.** (A) Split violin plots visualizing the expression levels of the indicated genes in fibroblast clusters identified by Kinchen et al. (2018) (GSE114374). Expression levels in normal colon fibroblasts are shown in green, and expression levels in DSS-induced inflammation-related fibroblasts in red. (B,C) qRT-PCR analysis of the indicated genes from isolated jejunum (B) and colon (C) from control mice and from mice 3, 24, and 72 h post 11 Gy WBI; *n*=3-4 mice per time point. Asterisks indicate statistical significance (*P<0.05, one-way ANOVA with Tukey's post hoc test). (D) Immunofluorescence staining of PDGFRA and NRG1 in regenerating (4 days post irradiation) mouse jejunum and colon. White dotted lines indicate the border between the stroma and the epithelium; yellow arrowheads indicate colocalization of NRG1 and PDGFRA. Scale bars: 50 µm. (E,F) qRT-PCR analysis of the indicated genes in primary intestinal fibroblasts with 24-h (E) and 72-h (F) treatments of EGF, EREG, NRG1 (100 ng/ml), LPS (1 μg/ml), TNFA, IFNG (10 ng/ml) and IL-1B (20 ng/ml). Data were derived from two to four independent mice with at least two technical replicates per mouse. Asterisks denote statistically significant (*P<0.05) differences from non-treated fibroblasts (one-way ANOVA with Tukey's post hoc test). Bar graphs (B,C,E,F) depict the mean, and error bars represent s.d.

Dextran sodium sulfate (DSS) induces severe damage to the colonic epithelium followed by inflammation, and upon DSS removal, rapid epithelial regeneration occurs, including reprogramming into a fetal-like state (Yui et al., 2018). We set to address whether *Ereg* and *Nrg1* expression levels are altered in damage-activated fibroblasts by analyzing the scRNA-seq dataset containing data from fibroblasts isolated from healthy and DSS-treated mice colons (Kinchen et al., 2018). We observed three *Pdgfra*-expressing fibroblast clusters and named them Str1, Str2 and Str3/4 according to the original analysis (Kinchen et al., 2018). Of the established markers of fibroblast subsets, *Foxl1* and *F3*, as well as *Procr* (encoding CD201) (Melissari et al., 2022), were specifically expressed in the Str2 cluster (Fig. 2A; Fig. S2C). *Cd34* (Stzepourginski et al., 2017) was expressed in both Str1 and Str3/4 populations, and *Cd81* (McCarthy et al., 2020a) specifically in Str3/4 (Fig. S2C). Consistently with the original analysis, *Il11* and *Cxcl13* were upregulated in inflammatory conditions in all subpopulations, whereas *Il33* baseline expression and induction were specific to Str3/4 (Kinchen et al., 2018) (Fig. S2D). Of the EGF family ligands, *Ereg* was expressed in non-inflamed conditions in both Str1 and Str2 clusters in this dataset, whereas *Nrg1* was specific to the Str2 cluster. In the inflamed fibroblasts, *Ereg* and *Nrg1* expression was increased, and the expression pattern expanded to other fibroblast populations (Fig. 2A). Other EGF family ligands showed no or minor expression in the fibroblast populations except for *Btc*, which was induced upon DSS treatment in the Str1 population, indicating that BTC may also represent a fibroblast-derived EGF family ligand in inflammatory conditions (Fig. S2E). These data indicate that *Ereg* and *Nrg1* expression is induced by inflammatory conditions in GI fibroblasts.

High-dose whole-body irradiation (WBI) leads to acute death of cycling cells, including the Lgr5-expressing ISCs, and the damaged epithelium then undergoes a highly proliferative regenerative stage at 3-4 days post irradiation (Ayyaz et al., 2019; Tian et al., 2011). To address the expression levels of *Ereg* and *Nrg1* in regenerating tissue *in vivo*, we applied qRT-PCR to jejunum and colon tissues at 3, 24 and 72 h after 11 Gy γ-WBI. *Egf*, the stem cell marker *Lgr5*, and the proliferation marker *Mki67* were also included in the analysis. *Egf* expression did not significantly change in response to irradiation-induced injury, whereas *Ereg* and *Nrg1* were upregulated at 72 h post irradiation, at which point high *Mki67* expression indicated active proliferation (Fig. 2B,C). Similar expression dynamics were previously shown for *Nrg1* (Jardé et al., 2020). *Ereg* expression has been previously shown to be stromal but to be induced in the epithelium during regeneration (Gregorieff et al., 2015; Yui et al., 2018). Accordingly, we noted both stromal and epithelial induction of *Ereg* mRNA in the small intestine after irradiation (Fig. S2B). NRG1 expression was also increased but remained strictly stromal in the regenerative phase at 4 days post irradiation (Fig. 2D; Fig. S2B).

Next, we aimed to investigate the upstream regulation of the identified EGF family ligands in the intestinal fibroblasts. We cultured primary intestinal fibroblasts and treated them with lipopolysaccharide (LPS), a bacterial cell wall component mimicking bacterial invasion, as well as the proinflammatory cytokines tumor necrosis factor α (TNFA, encoded by *Tnf*), interferon γ (IFNG), and interleukin 1β (IL-1B), which are rapidly released from multiple cell types upon inflammation to initiate innate and adaptive immune responses and tissue regeneration. In addition, we treated the cells with recombinant EGF, EREG and NRG1 to test for potential feedback loops of the EGF family ligands in this assay. As expected, LPS, TNFA and IFNG led to increased expression of *Tnf* in the fibroblasts, confirming that the cultured primary intestinal fibroblasts retain their responsiveness to inflammatory pathways (Fig. 2E). LPS and TNFA also induced a significant increase in *Ereg* expression, indicating that inflammatory signals activate *Ereg* transcription in fibroblasts. *Ereg* mRNA was also induced approximately twofold by EGF and EREG treatments, albeit the EREG treatment did not reach statistical significance (*P*=0.0578). Surprisingly, none of the tested conditions induced *Nrg1* expression (Fig. 2E), suggesting that unidentified upstream regulators induce *Nrg1* expression from fibroblasts, or that the stiff cell culture conditions induce *Nrg1* expression in primary intestinal fibroblasts without additional upstream activators. Interestingly, however, *Nrg1* expression was downregulated upon prolonged (72 h) culture with EGF and EREG, suggesting a negative feedback loop (Fig. 2F).

### NRG1 promotes *de novo* crypt formation and protects intestinal organoids from irradiation-induced damage

**Fig. 3.**
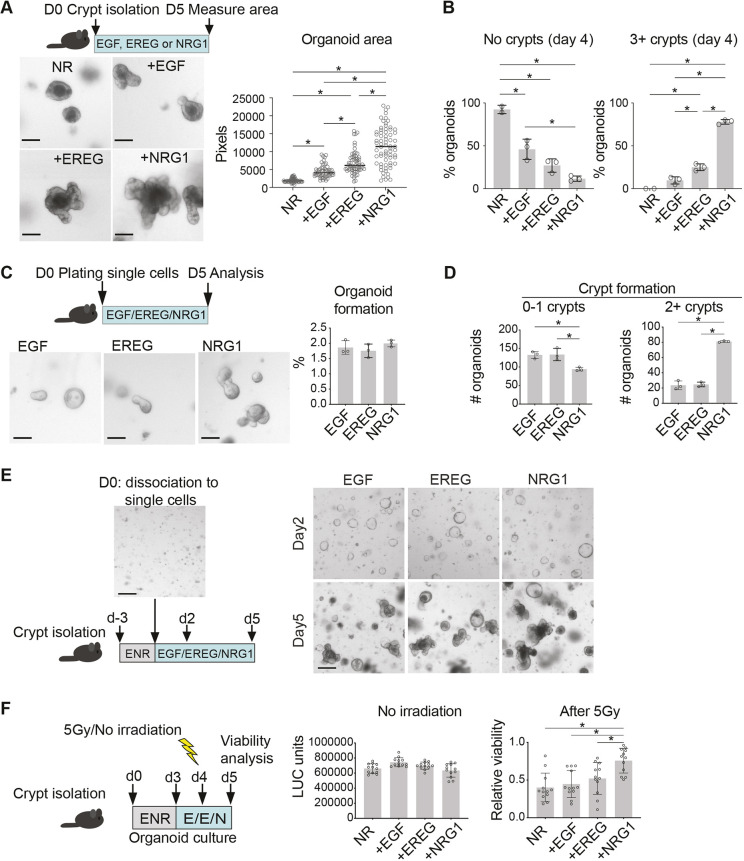
**NRG1 enhances crypt budding and protects intestinal organoids from irradiation-induced damage.** (A) Outline of the experiment, representative images and quantification of the small intestinal organoid area on day 5 in culture. NR, NOGGIN and RSPO1. Scale bars: 100 µm. (B) Percentage of organoids with zero (no crypts) or at least three (3+ crypts) *de novo* crypt budding events on day 4 in culture. (C) Approximately 5000 single EGFP^+^ cells dissociated from *Lgr5-Cre^ERT2^;IRES-EGFP* mouse small intestinal epithelium were plated per well and cultured with EGF, EREG or NRG1. Representative images and quantification of organoid formation capacity at day 5 post plating from three independent experiments are shown. Scale bars: 100 µm. (D) Organoid budding frequency at day 5 after plating. Three parallel wells per condition were analyzed and a representative of three independent experiments is shown. (E) Organoids were cultured for 3 days in ENR, dissociated into single cells and replated in EGF, EREG or NRG1 (100 ng/ml)-containing media. Representative images of two independent experiments of day 2 and day 5 culture from each condition are shown. Scale bars: 200 µm. (F) Luciferase (LUC)-based viability assay of irradiated small intestinal organoids grown in the presence of EGF, EREG and NRG1 (E/E/N; 100 ng/ml). Left graph: viability of non-irradiated control organoids on day 5. Right graph: viability of organoids 24 h after 5 Gy γ irradiation relative to the control (non-irradiated) organoids. A representative of three independent experiments is shown. Asterisks denote a statistically significant difference (*P<0.05; one-way ANOVA with Tukey's post hoc test). Bar graphs (A-D,F) depict the mean, error bars represent s.d.

In *ex vivo* culture of the primary intestinal epithelium, individual stem cells or crypts develop into three-dimensional organoids with multiple crypt domains, paralleling *in vivo* epithelial regeneration in which crypt fission enables new crypt formation (Sprangers et al., 2021). *De novo* crypt formation in the organoids requires both remodeling of the apical F-actin (Hartl et al., 2019) and a transient wave of YAP pathway activation (Serra et al., 2019). Organoids are also used to model intestinal epithelial responses to irradiation-induced damage (Montenegro-Miranda et al., 2020). To assess the functional role of NRG1 and EREG in organoid cultures, we compared their epithelial growth-supporting efficiency with EGF (Fig. 3A). As expected, crypts with no EGF family ligands failed to form budding organoids and remained round and cyst like, whereas the supplemented cultures started budding new crypt units in culture. EREG was slightly more efficient than EGF in inducing crypt budding and organoid size compared to EGF, whereas NRG1 enhanced crypt budding and organoid size remarkably (Fig. 3A,B). This was consistent with a previous report showing increased cell number in organoids treated with NRG1 compared to that in organoids treated with EGF (Jardé et al., 2020). The increased organoid area was evident at concentrations of 10 ng/ml and 100 ng/ml (Fig. S3A).

Interestingly, a previous study reported that NRG1 was able to maintain established human fetal intestinal organoids but failed to induce the growth of the epithelium when it was dissociated to single cells (Holloway et al., 2021). To address the impact of NRG1 compared with EGF and EREG in supporting the clonogenicity of Lgr5^+^ ISCs, we cultured EGFP^+^ cells from *Lgr5-EGFP-IRES-Cre^ERT2^* mice (Barker et al., 2007). Although the organoid-forming capacity was similar among the treatments, NRG1 significantly enhanced the organoid crypt budding compared to that seen upon EGF and EREG treatment (Fig. 3C,D). In addition, unlike in human fetal organoids, NRG1 supported *de novo* crypt formation in the intestinal epithelium *ex vivo* after passaging (Fig. 3E). In summary, these data show that both EREG and NRG1 can substitute for EGF in the adult mouse intestinal epithelium organoid and single-cell growth medium, and that exogenous NRG1 promotes dramatically increased *de novo* crypt formation in the intestinal epithelium *ex vivo*.

Given the increased expression of *Ereg* and *Nrg1* in the small intestine and colon following irradiation, and previous reports indicating increased intestinal proliferation in NRG1-treated mice upon irradiation (Jardé et al., 2020), we tested the efficiency of EGF, EREG and NRG1 in protecting the intestinal epithelial organoids from irradiation-induced damage. Notably, this approach allows focusing on the direct effects of the exogenous growth factors in the isolated epithelium. To exclude long-term culture-induced changes in organoid size (Fig. 3A,B), we first cultured all organoids in the presence of the organoid growth-supporting ENR medium (see Materials and Methods) for 3 days, followed by EGF, EREG or NRG1 treatment for 48 h. Then, 5 Gy γ-irradiation was performed 24 h before viability measurements (Fig. 3F). This treatment scheme did not induce significant differences in the viability of non-irradiated organoids, but the NRG1-treated organoids were significantly protected from irradiation-induced loss of viability, indicating that exogenous NRG1 functionally contributes to intestinal epithelium integrity upon irradiation-induced damage (Fig. 3F). This was associated with reduced apoptotic activity (Fig. S4).

Although EGF and EREG are known to bind and signal through EGFR, NRG1 binds ERBB3, which lacks the intracellular signaling domain, and signals predominantly via ERBB2, lacking the ligand-binding domain (Wieduwilt and Moasser, 2008). The ERBB2/3 heterodimer is a potent inducer of the PI3K-AKT pathway (Ruiz-Saenz et al., 2018), suggesting that differences in downstream pathway activation could explain the differential effects of NRG1 compared with those of EGF and EREG. Indeed, we noted that incubation of freshly isolated intestinal epithelium with NRG1 induced a robust phosphorylation of AKT, whereas MAPK pathway activation marked by ERK1/2 phosphorylation was strongest in the EGF-treated epithelium (Fig. S3B). NRG1 also induced strong phosphorylation of the ERBB3 receptor, whereas EGFR was activated mostly by EGF (Fig. S3B). The more modest EGFR-ERK1/2 response by EREG likely reflects the reported low-affinity binding of EREG to EGFR, inducing weaker but prolonged signaling compared to EGF (Freed et al., 2017). Next, we addressed the requirement of active EGFR and ERBB2 signaling to organoid viability in EGF-, EREG- and NRG1-treated organoids using specific inhibitors. The EGFR inhibitor gefitinib reduced organoid viability in all culture conditions, but the ERBB2 inhibitor tucatinib reduced viability specifically in the NRG1-treated organoids (Fig. S3C,D), indicating that signaling through EGFR is essential for organoid viability in all conditions, but ERBB2 signaling is specifically required for the NRG1-induced growth. Taken together, these data show that NRG1 signals via ERBB2/3, activates the AKT pathway, promotes intestinal epithelium growth and protects the epithelium from irradiation-induced damage.

### NRG1 treatment induces a fetal/regenerative transcriptional program in the intestinal epithelium

**Fig. 4.**
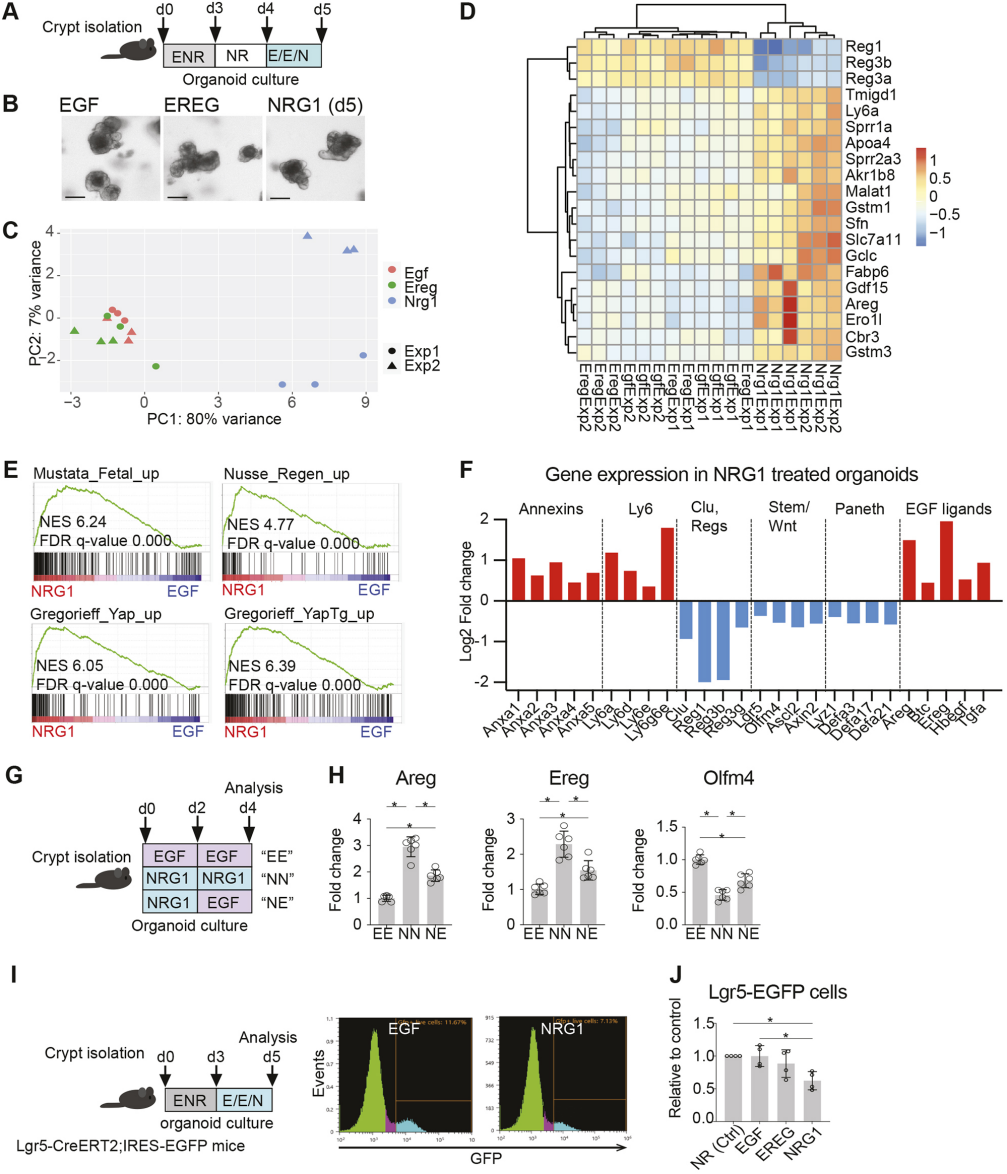
**NRG1 induces a fetal/regenerative transcriptional program in the intestinal epithelium.** (A-F) RNA sequencing of small intestinal organoids grown with EGF, EREG or NRG1. (A) Outline of the experiment. RNA was isolated at day 5 and submitted for sequencing. E/E/N, EGF/EREG/NRG1 (100 ng/ml). (B) Representative images of organoids treated with EGF, EREG and NRG1 for 24 h. Scale bars: 100 µm. (C) Principal component analysis (PCA) plot of EGF-, EREG- and NRG1-treated organoid transcriptomes. (D) Unsupervised hierarchical clustering heatmap of the individual samples sequenced. The top 20 genes driving the differences are shown. (E) GSEA of the log_2_FC-ranked gene list of NRG1- versus EGF-treated organoids. (F) Gene expression shown as log_2_FC of the indicated genes in NRG1- compared to EGF-treated organoids. Only genes with adjusted *P*-value<0.1 are shown. (G,H) Expression of *Areg*, *Ereg* and *Olfm4* in small intestinal organoids for 4 days with EGF, NRG1 or a pulse of NRG1 followed by EGF. (G) Experimental outline. (H) Expression level of indicated genes at day 4. Data are combined from two independent experiments. (I,J) Number of Lgr5-EGFP cells in small intestinal organoids treated with EGF, EREG or NRG1. (I) Experimental setup and representative plots of the flow cytometry analysis. (J) Relative amounts of Lgr5-EGFP cells in organoids treated with EGF, EREG, or NRG1 from four independent experiments. Ctrl, no EGF ligand added. Asterisks indicate statistically significant differences (*P<0.05; measured by one-way ANOVA with Tukey's post hoc test). Bar graphs (H,J) depict the mean, error bars represent s.d.

To get further insight into the functional differences of EGF-, EREG- and NRG1-induced signaling in the intestinal epithelium, we performed RNA sequencing. To exclude the size differences observed in long-term cultures (Fig. 3A,B) we first cultured intestinal organoids for 3 days in ENR medium, removed exogenous EGF for 24 h and then added EGF, EREG or NRG1 to the culture medium for 24 h before RNA isolation (Fig. 4A,B). Principal component analysis (PCA) revealed that the transcriptomes of the EGF- and EREG-treated organoids clustered together, whereas the transcriptomes of the NRG1-treated organoids separated along principal component (PC) 1, explaining 80% of the variance between samples and indicating that NRG1 induces a markedly distinct gene expression signature compared to EGF and EREG (Fig. 4C; Table S1). The NRG1-treated samples also clustered together in the hierarchical clustering analysis (Fig. 4D). Interestingly, *Ly6a*, *Sprr1a* and *Areg*, previously shown to be induced in regenerating and fetal GI epithelia (Nusse et al., 2018; Ohara et al., 2022; Yui et al., 2018), were among the most upregulated genes in the NRG1 cluster (Fig. 4D). Indeed, gene set enrichment analysis (GSEA) confirmed that the NRG1-induced transcriptome was significantly enriched with the fetal (Mustata et al., 2013) and regenerating (Nusse et al., 2018) intestinal epithelium gene sets. In addition, high enrichment was seen with YAP-induced intestinal epithelial gene sets (Gregorieff et al., 2015), consistent with the reported role of YAP in mediating intestinal regeneration and promoting a fetal-like transcriptome (Gregorieff et al., 2015; Ohara et al., 2022; Yui et al., 2018) (Fig. 4E). In agreement with previous data (Jardé et al., 2020), the NRG1-treated organoids partially survived treatment with verteporfin, which prevents the YAP/TAZ-TEAD interaction (Fig. S5A). However, YAP S127 phosphorylation was not reduced in the NRG1-treated intestinal epithelium, suggesting that the nuclear translocation regulated by S127 phosphorylation is not directly regulated by NRG1 (Fig. S5B). Instead, the enrichment of YAP signature (Fig. 4E) could be at least in part mediated by the NRG1-induced reduction of VGLL4 (Fig. S5C,D), an inhibitor of TEAD signaling that was recently shown to be a major repressor of YAP/TEAD-induced transcription (Cai et al., 2022).

Of note, the NRG1-induced induction of regenerative signaling was not associated with spheroid morphology reported previously in the context of fetal-like phenotypes (Mustata et al., 2013; Roulis et al., 2020; Yui et al., 2018), ruling out the possibility that the organoid morphology underlies the transcriptional responses. The NRG1-treated organoids remained dependent on RSPO1 unlike fetal organoids, which survive without it (Fordham et al., 2013), indicating that the fetal/regenerative transcriptomic conversion does not convert the organoids to entirely fetal-like organoids (Fig. S5E). Furthermore, NRG1-treated organoids were not able to grow and stayed cystic in type I collagen matrix, which has been reported to promote expression of fetal/regenerative genes (Yui et al., 2018) (Fig. S5F).

The transcriptional changes in the regenerating intestinal epithelium include enhanced expression of annexin and Ly6 family genes, upregulated expression of the revival stem cell marker clusterin (*Clu*) (Ayyaz et al., 2019), as well as reduced Wnt signaling, leading to a transient suppression of Lgr5^+^ stem cells and Paneth cells (Gregorieff et al., 2015; Nusse et al., 2018; Yui et al., 2018). Indeed, in our analysis, several annexins and Ly6 genes were upregulated, whereas the Wnt target genes as well as stem cell markers were downregulated (Fig. 4F). Surprisingly, however, expression of *Clu* was downregulated, indicating that not all features of the fetal/regenerative transcriptome were induced by NRG1. The reduction of Paneth cell markers (Fig. 4F) was consistent with a previous study demonstrating that ERBB3 signaling negatively regulates Paneth cell numbers through PI3K signaling (Almohazey et al., 2017). The antimicrobial genes *Reg1*, *Reg3b* and *Reg3a*, frequently upregulated in inflammatory conditions in a STAT3-dependent manner (Chen et al., 2019), were downregulated by NRG1, suggesting that differential triggers activate regenerative and inflammatory epithelial responses. Enteroendocrine cell-specific genes (Haber et al., 2017) were not consistently changed by NRG1, but Goblet cell-specific transcripts appeared to be downregulated (Fig. S6A,B), suggesting that NRG1 reduces Goblet cell differentiation. Interestingly, several EGF family ligands, especially *Ereg* and *Areg*, were upregulated upon NRG1 treatment (Fig. 4F), consistent with previous data showing their induction in the regenerating intestinal epithelium (Gregorieff et al., 2015; Yui et al., 2018). The NRG1-induced expression of *Ereg* and *Areg* as well as downregulation of the stem cell marker *Olfm4* were confirmed by qRT-PCR in continuous and pulsed NRG1 treatments (Fig. 4G,H), and these changes were reversed by blocking the ERBB2 signaling using tucatinib (Fig. S3E), suggesting a dynamic control of EGF family ligand signaling during the regenerative process orchestrated by stroma-derived NRG1.

Next, we addressed whether the reduction in the mRNA levels of *Lgr5* and *Olfm4* in NRG1-treated organoids reflected a reduced number of the Lgr5-expressing stem cells. We grew organoids from intestinal crypts isolated from *Lgr5-EGFP-IRES-Cre^ERT2^* mice for 3 days in ENR medium, followed by a 48-h incubation in the presence of EGF, EREG or NRG1, and analyzed the Lgr5-EGFP^+^ cell percentage by flow cytometry (Fig. 4I). The control organoids cultured with NOGGIN and RSPO1 (NR) survived for 48 h of culture without exogenous EGF, and the addition of EGF or EREG did not result in significant changes in the Lgr5-EGFP^+^ cell counts. However, NRG1 treatment reduced the Lgr5^+^ cell numbers by ∼40% (Fig. 4J). This analysis demonstrated that NRG1 treatment leads to a reduced number of the Lgr5^+^ ISCs, a feature previously connected to the regenerating epithelium (Gregorieff et al., 2015). Collectively, our data show that NRG1 induces a fetal/regenerative transcriptome in the intestinal epithelium, including induction of epithelial *Ereg* and *Areg* expression and reduction of the numbers of Lgr5^+^ ISCs.

### NRG1 remodels the epithelial actin cytoskeleton

**Fig. 5.**
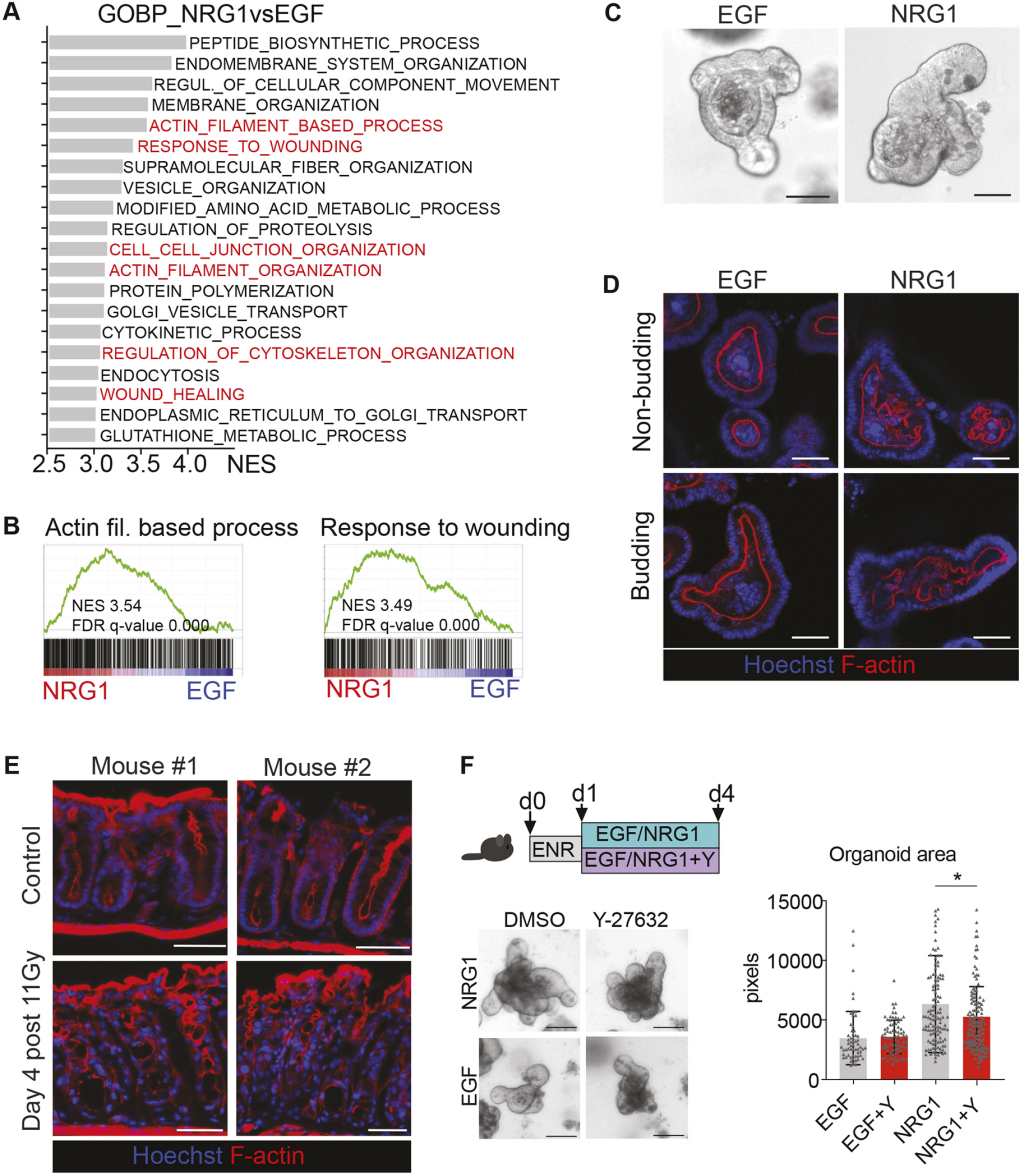
**NRG1 induces alterations in the epithelial actin cytoskeleton.** (A) GSEA of GO Biological Process (GO_BP) gene sets enriched in NRG1-treated small intestinal organoids. The top 20 enriched datasets are shown, and the cytoskeleton and wound healing-related datasets are highlighted in red. (B) GSEA plots of the NRG1 treatment-enriched GO_BP gene sets ‘Actin filament-based process’ and ‘Response to wounding’. (C) Representative images of small intestinal organoid morphology under a light microscope when cultured with EGF or NRG1. (D) Confocal microscopy analysis of apical F-actin in organoids grown for 48 h in the presence of EGF or NRG1. (E) Representative figure of F-actin staining in the distal colon from control and irradiated mice. (F) Effect of ROCK inhibition to small intestinal organoid growth. Representative images and quantification of the organoid area in the indicated conditions are from three independent experiments. Y, 10 µM Y-27632. Scale bars: 100 µm (C,F); 50 µm (D,E). The asterisk indicates a statistically significant difference (*P<0.05; measured by one-way ANOVA with Tukey's post hoc test). Mean and standard deviation are shown.

Unbiased GSEA analysis of NRG1-treated organoids against commonly used databases of biological pathway gene sets revealed positive enrichment for genes involved in PI3K/AKT/mTOR/HIF1A/hypoxia pathways, cell adhesion- and cytoskeleton-related pathways, metabolic and immune pathways, as well as tyrosine kinase receptor pathways (Fig. S7). As changes in cell adhesion and actin cytoskeleton underlie intestinal regenerative and wound healing processes (Ohara et al., 2022; Ubelmann et al., 2013; Yui et al., 2018), and because a similar analysis of Gene Ontology (GO) biological processes revealed several cytoskeleton- and wound healing-related signatures among the most significantly enriched GO terms in the NRG1 dataset (Fig. 5A,B), we set to study the actin cytoskeleton of NRG1-treated organoids. In bright-field microscopy, the NRG1-treated organoids presented with a typical bloated morphology (Fig. 5C). Phalloidin staining revealed that in EGF-treated organoids, F-actin was smoothly aligned with the apical side of the organoids, whereas NRG1 treatment resulted in a branching, irregular shape of the F-actin belt (Fig. 5D), reminiscent of the branching events reported to precede crypt fission in organoids (Langlands et al., 2016). Similar observations were made in the regenerating colonic epithelium *in vivo* 4 days after 11 Gy WBI (Fig. 5E). These data suggest that NRG1-treated organoids acquire a phenotype resembling crypt fission events, an event crucial for epithelial regeneration (Malipatlolla et al., 2019).

Rho-associated protein kinase (ROCK) phosphorylates the myosin light chain (MLC) subunit of non-muscle myosin, thereby promoting constriction of intracellular actin fibers. We assessed the requirement of actin constriction to NRG1-induced organoid growth by culturing the organoids in the presence of the ROCK inhibitor Y-27632. Although EGF-treated organoids grew similarly in the presence or absence of the ROCK inhibitor, NRG1-induced growth was partially compromised in the presence of Y-27632 (Fig. 5F), suggesting that ROCK activation-mediated changes in the actin cytoskeleton are involved in the regenerative, growth-promoting effects of NRG1. Collectively, these data indicate that NRG1 treatment induces regeneration-like changes in the morphology and in the actin cytoskeleton of intestinal organoids and that the NRG1-induced growth effect is partially mediated by ROCK activity.

### NRG1 does not promote tumorigenic growth of the intestinal epithelium

**Fig. 6.**
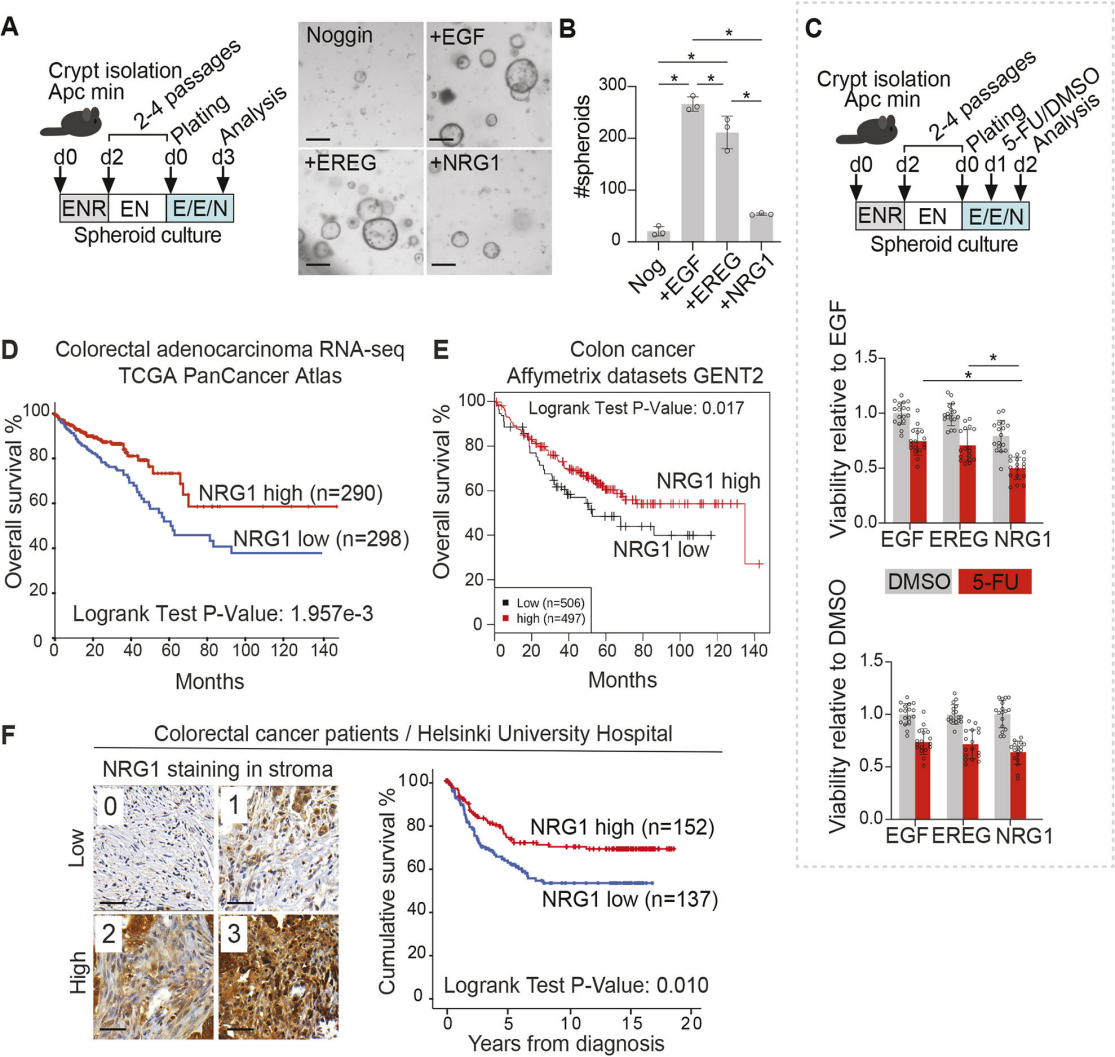
**NRG1 does not support the growth of tumorigenic epithelium.** (A,B) Culture of pre-tumorigenic *Apc* mutant small intestinal spheroids in media supplemented with EGF, EREG or NRG1. After initial culture in ENR, small intestinal organoids were passaged without RSPO1 (‘EN’) to select for *Apc* mutant spheroids. Representative images are shown (A). Scale bars: 200 µm. Quantification (B) of the number *Apc* mutant spheroids per well for the indicated conditions. Asterisks indicate statistical significance (*P<0.05; one-way ANOVA with Tukey's post hoc test); three parallel wells were counted per treatment. The experiment shown is a representative of three independent experiments. (C) Luciferase-based viability assay of *Apc* mutant small intestinal spheroids at 24 h after 5-FU treatment in the presence of EGF, EREG and NRG1. Relative viability at 24 h after addition of 5-FU or DMSO with indicated treatments is shown. Five independent experiments were conducted with two to six technical replicates (wells) each. Asterisks indicate statistical significance (*P<0.05; one-way ANOVA with Tukey's post hoc test). Bar graphs depict the mean, error bars represent s.d. E/E/N: EGF/EREG/NRG1 (100 ng/ml). (D) Kaplan–Meier curve depicting the probability of overall survival in CRC patients displaying high (>median) or low (<median) expression of *NRG1* mRNA assessed by RNA sequencing. Data were retrieved from the TCGA PanCancer Atlas. (E) Kaplan–Meier curve depicting the probability of overall survival in CRC patients displaying high (>median) or low (<median) expression of *NRG1* mRNA assessed by Affymetrix microarray analysis from multiple datasets collected in the GENT2 database. (F) Representative images of stromal NRG1 staining from CRC tissue sections, and Kaplan–Meier curve depicting the probability of disease-specific survival in CRC patients displaying high (score 2 to 3) or low (score 0 to 1) expression of stromal NRG1. Scale bar: 50 µm.

A recent study showed that fibroblast-derived PGE2 both promoted intestinal epithelial regeneration and enhanced tumorigenesis, demonstrating that regenerative stromal signaling may also contribute to tumor formation (Roulis et al., 2020). Interestingly, we observed increased mRNA expression of both *Ereg* and *Nrg1* in intestinal tumors of *Apc^min^* mice, which model early CRC by developing multiple adenomas throughout the length of the intestine, owing to Apc loss-mediated constitutively active Wnt signaling (Jackstadt and Sansom, 2016) (Fig. S8A). To address the functional relevance of this finding, we tested the growth-promoting ability of NRG1 and EREG compared to that of EGF in tumorigenic intestinal organoids from *Apc^min^* mice. We selected for biallelic *Apc* mutations by omitting RSPO1 from the culture medium (Fig. 6A), resulting in a spheroid morphology of early tumorigenic organoids as noted before (Langlands et al., 2018). Surprisingly, we noted that although EGF and EREG supported the growth of the tumorigenic spheroids, NRG1 failed to induce growth above the control levels (Fig. 6B), a result strikingly different from the superior growth-promoting effect of NRG1 in wild-type (WT) organoids (Fig. 3A,B). Previously, the *Apc* mutant organoids were passaged using enzymatic dissociation to single cells and NRG1 was shown to fail to initiate the growth of single-cell-dissociated human fetal intestinal organoids (Holloway et al., 2021). To test whether NRG1 would support the growth of *Apc* mutant organoids passaged without single-cell dissociation, we applied mechanical dissociation with pipetting, resulting in breaking the spheroids into fragments but not into single cells. This approach resulted in more robust growth of the organoids even without any EGF ligands, yet the NRG1 treatment failed to induce growth above control levels (Fig. S8B). AKT pathway activation occurred in organoids irrespective of *Apc* mutation, demonstrating that the *Apc* mutant organoids also responded to NRG1 treatment (Fig. S8C); however, this was not sufficient to support the growth to the levels induced by EGF and EREG. In addition, NRG1 did not protect the tumorigenic epithelium from radiation-induced damage (Fig. S8D). These results indicate that despite the increased expression of *Nrg1* in early tumor stroma, the growth-stimulating and protective effects of NRG1 are restricted to the WT epithelium, suggesting that suppression of Wnt signaling may be required for the NRG1 growth-promoting function.

To test how NRG1 treatment affects response to a chemotherapeutic drug, *Apc* mutant organoids were treated with 5-fluorouracil (5-FU) which induces acute toxicity in the intestinal epithelium *in vivo* and in organoids *ex vivo* (Rodrigues et al., 2021) (Fig. 6C). Interestingly, 5-FU treatment significantly reduced the viability of NRG1-treated *Apc* mutant organoids, demonstrating that NRG1 did not protect organoids from 5-FU-mediated toxicity (Fig. 6C). In WT organoids, the response to 5-FU was not significantly different following EGF, EREG and NRG1 treatments (Fig. S8E).

As loss of the tumor suppressor *APC* is a common and early feature of human CRC, we next set to analyze whether NRG1 expression correlates with survival in CRC. We first analyzed the overall survival probability of 588 CRC patients from The Cancer Genome Atlas (TCGA) Pan-Cancer Atlas with available RNA sequencing data (https://www.cancer.gov/tcga) using cBioPortal (https://www.cbioportal.org/) (Cerami et al., 2012; Gao et al., 2013). Intriguingly, high (above median) expression of *NRG1* predicted significantly improved overall survival in CRC (Fig. 6D). Similar results were observed in independent CRC cohorts by analysis of the GENT2 database (Park et al., 2019) (Fig. 6E). Interestingly, the significantly improved survival in CRC patients with high *NRG1* mRNA levels was observed in patients with higher than median levels of the expression of *ERBB3*, encoding a NRG1 receptor, suggesting that functional NRG1-ERBB3 signaling may underlie the beneficial prognosis in CRC (Fig. S9A). *ERBB3* mRNA level itself was not associated with altered prognosis (Fig. S9B). Of the other EGF family ligands, *TGFA* mRNA expression correlated with reduced overall survival (Fig. S9C), but other ligands did not correlate with significant survival differences (*EGF* and *EREG* shown in Fig. S9D, other ligands not shown).

To address whether the improved survival observed in CRC with high *NRG1* mRNA levels relates to stromal expression, we scored 315 CRC patients' tissue microarrays for NRG1 expression separately in cancer (epithelial) and in stromal cells. Out of 315 samples, 293 samples (93.0%) contained sufficient tumor tissue to allow evaluation of the NRG1 staining in cancer cells, and 289 samples (91.7%) were suitable to address the stromal expression. Interestingly, high stromal NRG1 expression correlated with significantly higher disease-specific survival (Fig. 6F), whereas the NRG1 expression level in cancer cells did not associate with survival differences (Fig. S9E). These data imply that high stromal expression of NRG1 is associated with improved disease specific survival in CRC.

## DISCUSSION

In this work, we set to investigate the roles of fibroblast-derived EGF family ligands in the intestinal epithelium. We identified EREG and NRG1 as the main fibroblast-derived EGF ligands, of which NRG1 acts as an upstream inducer of the fetal-like transcriptional reprogramming that occurs during intestinal regeneration. In homeostatic conditions, *Ereg* was expressed primarily from fibroblasts, but its expression was induced in epithelial organoids in response to the NRG1 pulse along with another EGF ligand, *Areg*. The induction of epithelial *Ereg* expression during inflammation and regeneration has been reported (Gregorieff et al., 2015; Serra et al., 2019; Yui et al., 2018), but the upstream activators have not been identified. Our finding suggesting that stromal NRG1 may act upstream of the epithelial EREG and AREG expression implies a dynamic interplay for the different EGF family ligands during homeostatic and damage repair. Our data agree with the previous report in which *Ereg* expression is expressed mostly in the stroma during homeostasis, and epithelial *Ereg* expression is induced upon inflammation (Gregorieff et al., 2015; Yui et al., 2018). Interestingly, *Ereg* was previously shown to be essential for colonic regeneration after inflammation (Lee et al., 2004), suggesting that the NRG1-induced *Ereg* expression may represent a critical step in the regenerative process. Thus, it will be interesting to address whether the NRG1-induced epithelial regeneration noted here and the increased epithelial proliferation upon damage reported previously (Jardé et al., 2020) requires induction of epithelial EREG expression. Interestingly, we noted that *Ereg* expression was induced by inflammatory agents in cultured intestinal fibroblasts. This increase of *Ereg* in stromal cells could result from the initial inflammatory environment of the damaged tissue and contribute to early tissue dynamics. We also discovered that EREG treatment led to downregulation of *Nrg1* mRNA expression in primary intestinal fibroblasts, suggesting a feedback loop ensuring that the injury-induced increase in NRG1 expression is resolved upon increased EREG expression.

Regenerating the intestinal epithelium changes its cytoskeletal organization and cell adhesion properties. Additionally, NRG1 treatment induced profound transcriptomic changes in the cytoskeleton and cell adhesion pathways and altered the epithelial apical F-actin belt organization, and the NRG1-mediated increase of organoid size was partially dependent on active ROCK signaling, suggesting that NRG1-mediated actomyosin constriction is involved in the regenerative response. Interestingly, stretching the epithelium by organoid inflation was recently shown to result in fetal/regenerative gene expression, loss of Lgr5^+^ stem cells and crypt fission (Tallapragada et al., 2021), confirming that mechanical cues play a role in epithelial reprogramming. Interestingly, EGFR family receptors complex with integrins on the cell surface to promote intracellular signaling (Ieguchi et al., 2010; Javadi et al., 2020; Kanakry et al., 2007; Morello et al., 2011). Thus, NRG1 binding may affect the actomyosin cytoskeleton via ERBB2/ERBB3/integrin-mediated activation of ROCK, presumably via activation of focal adhesion kinase (FAK) as suggested by our transcriptomic analysis. Indeed, FAK has been shown to be required for intestinal regeneration after irradiation (Ashton et al., 2010).

Another feature of the effect of NRG1 on epithelial signaling was the suppression of the WNT pathway and diminished numbers of Lgr5-expressing stem cells, consistent with previous work showing that epithelial regeneration requires temporary stem cell quiescence (Gregorieff et al., 2015). Interestingly, a previous study (Jardé et al., 2020) reported increased stemness-related gene expression in the intestinal epithelium in response to recombinant NRG1. This possibly reflects the differences in the experimental setup: although our data derived from organoids in which all epithelial cell types were represented, Jardé et al. (2020) compared the transcriptomics between isolated Lgr5^+^-high stem cells, Lgr5^+^-medium cells, Lgr5^+^-low progenitors and Lgr5-negative non-stem cells. Although the more sophisticated analysis by Jardé et al. (2020) allowed for comparisons between the distinct cell fractions, the relative contributions of the cell types may have been masked in those analyses.

Two recent articles identified NRG1 as a key ISC niche factor in the fetal human intestine (Holloway et al., 2021; Yu et al., 2021). Interestingly, NRG1 was shown in both reports to support the growth of fetal human intestinal organoids, but Holloway et al. (2021) found that EGF-depleted, single-cell-dissociated organoids were not successfully initiated by NRG1, indicating that single fetal human stem cells may need EGF (or other EGFR-activating ligands) for clonogenicity (Holloway et al., 2021). Our data showed that single Lgr5^+^ mouse adult stem cells can develop into organoids without exogenous EGF in the presence of NRG1. This may reflect true independency of EGF signaling, or a sufficient carry-over of membrane-bound EGF ligands to induce the initial growth of the Lgr5^+^ cells, as suggested before for Wnt ligands (Pentinmikko et al., 2019). Regarding functional effects, both Yu et al. (2021) and Holloway et al. (2021) used scRNA-seq of EGF- and NRG1-treated organoids and showed that compared to EGF, NRG1 induced more Lgr5^+^ stem cell clusters (Yu et al., 2021) and clusters expressing markers for Goblet cells and enteroendocrine cells (Holloway et al., 2021), indicating increased differentiation. In our analysis, stem and Goblet cell markers were decreased by NRG1, and no significant changes were identified for enteroendocrine cells, indicating that the functional effects induced by NRG1 on mature (mouse) epithelia differ from those on human fetal epithelia.

Our finding that NRG1 treatment did not support the growth of *Apc* mutant pre-tumorigenic organoids was intriguing. Interestingly, although EGFR is frequently activated in CRC and EGFR inhibitors are effective in a subset of CRCs (Sakata and Larson, 2022), the contribution of the NRG1 receptor complex ERBB2/3 signaling is more unclear. ERBB2 amplifications occur in about 5% of metastatic CRC, resulting in signal activation independently of upstream ligands (Mohamed et al., 2022). Rare *NRG1* gene fusions have, however, been identified as potential oncogenic drivers in CRC (Jonna et al., 2019). In mouse models of intestinal tumorigenesis, deletion of *Erbb2* and *Erbb3* has been reported to both increase and decrease the tumor burden, depending on the genetic background (Rojas et al., 2021). Our data from two independent sources showed that high stromal NRG1 expression correlates with improved survival in CRC, suggesting that in most CRCs, high stromal NRG1 expression is in fact beneficial. Indeed, hypermethylation of the *NRG1* promoter was previously shown to occur in colorectal adenomas and cancer, suggesting tumor suppressive function of *NRG1* in CRC (Øster et al., 2011). Of note, however, mesenchymal NRG1 has also been reported to correlate with reduced progression-free survival in a previous study of 54 CRCs (De Boeck et al., 2013). Our results indicating that *Apc* mutant organoids were more responsive to the chemotherapeutic drug 5-FU in the presence of NRG1 than EGF suggest that high levels of NRG1 could be linked to benefits in clinical drug responses. This hypothesis would be an interesting topic for a follow-up study.

In summary, our work identifies fibroblast-derived NRG1 as an inducer of fetal/regenerative phenotype in the intestinal epithelium that protects WT organoids from damage but does not support the growth of the (pre)tumorigenic intestinal epithelium lacking the tumor suppressor gene *Apc*. This could open possibilities to test NRG1-based therapies in protecting normal tissue from injury induced by cytotoxic drugs or radiation treatment without accelerating tumorigenesis.

## MATERIALS AND METHODS

### Mice

Male and female *Lgr5-EGFP-IRES-Cre^ERT2^* mice (*Mus musculus*) (stock no. 008875) (Barker et al., 2007) and C57BL/6J-Apc^min^/J mice (stock no. 002020) from the Jackson Laboratory were used. Animals were housed at the University of Helsinki Laboratory Animal Center and Columbia University Medical Center according to national and international legislation and guidelines (license: ESAVI/8804/2020). Mice had free access to food and water. Male and female mice were used for intestinal crypt isolation at 2-4 months of age. WBI (11 Gy) was conducted with a cesium-137-based γ-ray irradiator Mark I (J. L. Shepherd & Associates, San Fernando, CA, USA) or OB29/4 (STS, Braunschweig, Germany). Post-interventional monitoring was performed according to Columbia University and Helsinki University animal protocols.

### Organoid and spheroid culture

Intestinal organoids were cultured with a modified version of a previously published protocol (Sato et al., 2009). The small intestine was removed, flushed with ice-cold PBS and opened longitudinally. After removal of extensive mucus, the intestine was cut into 2-3 mm fragments that were placed in a 50 ml tube with PBS on ice and shaken gently. The tissue fragments were let to settle to the bottom and the supernatant was replaced by 20 ml of 10 mM EDTA in PBS. The tube was placed on ice horizontally and shaken gently for 1 h 45 min and the buffer was changed three times during the first 45 min. To detach the crypts, the tube was shaken vigorously for 15 s, and the detached crypts were filtered through a 70 µm cell strainer and collected by centrifugation (200 ***g*** for 2 min). The pellet was washed with ice-cold PBS and collected as above. The crypts were resuspended in basal organoid medium, composed of Advanced Dulbecco's modified Eagle medium (DMEM):F12 (12634010, Gibco) supplemented with 1 M HEPES pH 7.4 (H0887, Sigma-Aldrich), 1× GlutaMAX (35050061, Gibco), 1× penicillin-streptomycin (P4333, Sigma-Aldrich), 1× N-2 supplement (17502001, Gibco) and 1× B-27 supplement (17504044, Gibco). The crypts in the basal medium were mixed in a 1:3 ratio with Matrigel (356231, Corning) before plating 20 µl domes to 48-well plates. The domes were overlaid with ENR medium, composed of basal organoid media supplemented with EGF (Gibco, PMG8041; 50 ng/ml), NOGGIN (Peprotech, 250-38; 100 ng/ml), R-Spondin 1 (R&D Systems 3474-RS; 250 ng/ml) and N-acetylcysteine (A0737, Sigma-Aldrich; 1 µM). Y-27632 (Y0503, Sigma-Aldrich; 10 µM) was included in the ENR medium for the first 2 days, except for the experiment shown in Fig. 5F in which all conditions contained Y-27632 only for the first day of the culture. In Fig. S5F, crypts were plated in a 1:3 ratio with type 1 collagen (354249, Corning), according to suggested coating procedures provided by Corning's Certificate of Analysis. The final concentration of type 1 collagen was 6 mg/ml. For flow cytometry analysis (Fig. 4I-J), single Lgr5-EGFP^+^ cells from organoid cultures were collected using organoid harvesting solution (3700-100-01, R&D Systems) and centrifuged at 200 ***g*** for 2 min. Organoids were dissociated with 1 mg/ml DNase 1 (10104159001, Roche) in TrypLE Express (12605-010, Thermo Fisher Scientific) for 1 min in a 37°C water bath, followed by trituration through a 1 ml pipette on ice 12 times. The dissociated cells were suspended into 1 ml of basal medium (see above) containing 10 µM Y-27632 and 1 µM N-acetylcysteine, filtered through a 40 µm strainer and spun down at 200 ***g*** for 5 min. The pellet was resuspended in the same medium as before with the addition of 1% bovine serum albumin (P6154-100GR, Biotop) and filtered again through a cell-strainer cap into a 5 ml tube (352235, Corning); 1 mM of EDTA was added to prevent cells from clumping. EGFP^+^ cells were sorted with a Sony SH800z cell sorter. For experiments comparing the effects of EGF family ligands, EGF (R&D Systems, 236-EG-200), NRG1 (R&D Systems, 5898-NR) and EREG (R&D Systems, 1195-EP-050) were used at 100 ng/ml. Gefitinib (sc-202166, 1 µM) was purchased from Santa Cruz Biotechnology. Tucatinib (HY-16069, 2 µM) was purchased from MedChemExpress. 5-FU (F6627, 10 µM) and verteporfin (SML0534, 1 µM) were purchased from Sigma-Aldrich. Irradiation to organoids were conducted with the cesium-137 γ-irradiator OB29/4 (STS).

For *Apc* mutant organoids, crypts were collected from 15- to 17-week-old tumor-bearing *Apc^min^* mice and, after the first 2 days, cultured without the addition of RSPO1 to select for biallelic *Apc* mutant growth. Organoids were imaged with an EVOS FL microscope (Thermo Fisher Scientific). The *Apc* mutant organoids were passaged by harvesting the organoids using ice-cold organoid harvesting solution. Organoids were collected into a tube and centrifuged at 300 ***g*** for 5 min. Organoids were dissociated mechanically by pipetting up and down 40-50 times with a 200 μl pipette or enzymatically with 1 mg/ml DNase 1 in TrypLE Express for 1 min in a 37°C water bath followed by trituration through a 1 ml pipette on ice 12 times. The dissociated cells were suspended into 5 ml of basal medium (see above) containing 10 µM Y-27632 and 1 µM N-acetylcysteine and spun down at 300 ***g*** for 5 min. Organoids were resuspended in the desired medium and mixed with Matrigel as described above.

### Isolation and culture of single Lgr5-EGFP cells

Intestinal crypts from *Lgr5-EGFP-IRES-Cre^ERT2^* mice were collected as detailed above. The crypts were dissociated to single cells using 1 mg/ml DNase 1 in TrypLE Express for 3 min in a 37°C water bath followed by trituration through a 1 ml pipette on ice for 12 times. The dissociated cells were suspended into 5 ml of basal organoid medium (see above) containing 10 µM Y-27632 and 1 µM N-acetylcysteine, filtered through a 40 µm strainer and spun down at 200 ***g*** for 5 min. The pellet was resuspended in the same medium as before with addition of 1% bovine serum albumin and filtered again through a cell-strainer cap into a 5 ml tube (352235, Corning); 1 mM of EDTA was added to prevent cells from clumping. EGFP^+^ cells were sorted with a Sony SH800z cell sorter. The gate was set using crypt cells isolated from control (non-EGFP-expressing) mice. After the sorting, cells were spun down at 200 ***g*** for 5 min and then at 400 ***g*** for 1 min, and plated to Matrigel domes containing 5000 EGFP^+^ cells each. Single cells were cultured with ENR medium supplemented with 1 µg/ml RSPO1 and 2.5 µM CHIR99021 (SML1046, Sigma-Aldrich) for the first 2-3 days.

### Tissue preparation and staining

Small intestinal and colon tissues were collected, washed with PBS and fixed in 4% paraformaldehyde in 4°C for 24 h. During the fixation, the paraformaldehyde was replaced once. The intestines were embedded in paraffin as Swiss rolls with a Sakura Tissue-Tek VIP 5 Jr tissue processing system. Then, 5 µm sections were cut using a microtome (Shandon AS325, Marshall Scientific). Paraffin-embedded sections were deparaffinized in a xylene and ethanol series. Antigen retrieval was performed using 1× Dako antigen retrieval solution at either pH 6 or pH 9 (S1699 and S2367, Agilent Technologies) at 95°C for 20 min, followed by a 30 min incubation at room temperature (RT). Permeabilization was performed with 0.1% Triton X-100 in TBS for 10 min at RT, followed by blocking for 30 min using 5% donkey serum in TBS. The samples were incubated with anti-NRG1 (ab191139, Abcam, 1:500) and anti-PDGFRA (AF1062, R&D systems, 1:100) antibodies overnight at 4°C. They were then incubated with secondary antibodies [Alexa Fluor 488 donkey anti-rabbit IgG (A21206, Thermo Fisher Scientific) and Alexa Fluor 594 donkey anti-goat IgG (A11058, Thermo Fisher Scientific)] were incubated in blocking buffer (1:500 dilution) for one hour at RT and washed three times for 5 min in TBS containing 0.1% Tween 20. Nuclei were stained with 10 µg/ml Hoechst 33324 (62249, Thermo Fisher Scientific) in PBS for 3 min. Slides were rinsed with TBS and water before mounting with Immu-Mount (9990402, Thermo Fisher Scientific). For frozen sections, the colon was fixed as above and placed in 30% sucrose in PBS overnight at 4°C. The next day, tissue pieces were embedded in OCT compound (4583, Sakura) and frozen. Then, 8 µm sections were cut and incubated in blocking buffer for 30 min at RT, followed by staining for 1 h at RT with Alexa Fluor 488 phalloidin (A12379, Thermo Fisher Scientific; 1:250 dilution in blocking buffer).

### Whole-mount staining

Organoids were grown in eight-chamber slides (354108, Corning) and fixed with 4% paraformaldehyde for 1 h at RT. After washing with PBS, the organoids were permeabilized with PBS containing 0.5% Triton X-100 for 30 min at RT and blocked with 10% normal goat serum in PBS containing 0.25% Triton X-100 for 60 min. Alexa Fluor 594 phalloidin (A12381, Thermo Fisher Scientific) was diluted in the blocking buffer (1:500) and incubated for 1 h at RT and washed once with PBS containing 0.25% Triton X-100, followed by three PBS washes. The nuclei were stained with 10 μg/ml Hoechst 33324 in PBS for 5 min and the slides were mounted with Mowiol (81381, Sigma-Aldrich).

### Fluorescence *in situ* hybridization

RNAscope Multiplex Fluorescent Reagent kit v2 (323100, ACDBio) was used according to the manufacturer's recommendations. The RNAscope probes (acquired from ACDBio) used were: RNAscope Probe Mm-Ereg (437981), RNAscope Probe Mm-Nrg1-Cust2-C3 (422961-C3), RNAscope Negative control probe-DapB (310043), RNAscope Negative control probe DAPb-C3 (310043-C3), RNAscope Positive Control Probe- Mm-Ubc (310771), RNAscope Positive control probe Mm-Ubc-C3 (310771-C3) and RNAscope Probe Diluent (300041). The Opal 570 Reagent Pack (FP1488001KT, Akoya Biosciences) fluorescent label (1:1000 dilution) was used to stain *Ereg* and *Nrg1* mRNAs.

### Imaging

Imaging was performed with a Leica TCS SP8 CARS confocal microscope and with Zeiss Axio Imager M2 microscope. Image analysis was conducted using ImageJ.

### Viability assays

Organoid viability was assayed with CellTiter-Glo 3D Cell viability assay (G9681, Promega) according to the manufacturer's protocol. The cell viability was measured with FLUOstar Omega plate reader (BMG Labtech).

### Apoptosis assay

The apoptosis levels of organoids were measured using Caspase-Glo 3/7 3D Assay (G9681, Promega) according to the manual's standard protocol for Matrigel-embedded microtissues. Mixing of the reagent with Matrigel was enhanced by pipetting the reagent ten times in each well. Caspase 3/7 levels were measured with FLUOstar Omega plate reader (BMG Labtech).

### Primary fibroblast culture

Primary intestinal fibroblasts were isolated using a modified version of a previously published protocol (Stzepourginski et al., 2017). The whole small intestine was washed with PBS, opened longitudinally and cut into 1-2 cm fragments. Tissue fragments were incubated in PBS containing 5 mM EDTA and 1 mM DTT for 20 min and placed horizontally in a rotating incubator at 37°C and 250 rpm. The EDTA solution was removed by washing the tissue pieces three times with ice-cold PBS by vigorously shaking for 15 s by hand, followed by foam removal and replacement of PBS between shakes. After the washes, the pieces were placed in PBS and minced thoroughly with a scalpel. The tissue pieces were placed in a collagenase digestion solution (2% fetal bovine serum in PBS with collagenase type II and IV; Thermo Fisher Scientific) and rotated at 250 rpm horizontally for 20 min at 37°C. Cold fibroblast growth medium (DMEM containing 20% fetal bovine serum, 1% penicillin-streptomycin and 1% L-glutamine) was added to stop the collagenase activity. Single cells were released from the digestion suspension by trituration, and the cells were pelleted by centrifugation for 10 min at 300 ***g*** and resuspended in 37°C growth medium. The suspension was plated on a Petri dish and cells were allowed to attach for 1 h at 37°C, after which the plate was washed three times with PBS to remove debris and unattached cells, and fresh medium was added to the culture. For *in vitro* experiments, approximately 150,000 primary fibroblasts were plated on six-well plates and allowed to attach overnight before starting the treatments indicated in the text. For each biological replicate, at least two technical replicates per treatment were included. TNFA (315-01A, 20 ng/ml), IL-1B (211-11B, 20 ng/ml) and IFNG (315-05, 10 ng/ml) were purchased from PeproTech. Lipopolysaccharide (LPS) (L4516, Sigma-Aldrich) was used at 1000 ng/ml.

### qRT-PCR

RNA from organoids was isolated using the Nucleospin RNA II isolation kit (Macherey-Nagel) and from primary fibroblasts with the TRI Reagent (AM9738, Thermo Fisher Scientific) according to the manufacturers’ recommendations. Complementary DNA (cDNA) was produced with TaqMan reverse transcription kit (N8080234, Applied Biosystems) according to the protocol, except that the final concentration of MgCl_2_ was 5 mM and the final concentration of MultiScribe reverse transcriptase was 1 U/µl. Reverse transcription reaction was done with MJ Research PTC-200 Thermal Cycler (Marshall Scientific). qRT-PCR was performed with KAPA SYBR FAST qPCR Master Mix Universal reagent (KK4618, KAPA Biosystems). All primers were diluted in nuclease-free water to a final concentration of 500 nM and a reaction volume of 16 µl. Samples were run with StepOnePlus Real-Time PCR System (Thermo Fisher Scientific). *Actb* was used as the internal control. The relative fold change was calculated using the ΔΔC_T_ method (Livak and Schmittgen, 2001), except for Fig. 1C for which the ΔΔC_T_ value was used to calculate the percentage of *Actb* expression (2^−ΔCT^×100). Primers used for qRT-PCR are given in Table S3.

### Western blotting

Small intestinal crypts were isolated as detailed above and suspended in basal organoid medium. The crypts were incubated for 20 min with 100 ng/ml EGF, EREG or NRG1 at 37°C, and for Fig. S8B, passaged intestinal organoids and *Apc*^min^ spheroids were cultured for 2 days before collection and treatments. Samples were lysed in 1× SDS lysis buffer (2% SDS, 60 mM Tris-HCl pH 6.8 and 10% glycerol) supplemented with 1× protease and phosphatase inhibitor cocktails (Roche). The protein concentrations were measured using a Bio-Rad DC protein assay and 10 μg of the lysates were run in 8% acrylamide gels and blotted to nylon membranes using the Fast Blotter (Bio-Rad). After Ponceau staining, the membranes were blocked with 5% milk in TBS containing 0.1% Tween 20 and incubated with primary antibodies overnight at 4°C. HRP-conjugated anti-rabbit or anti-mouse secondary antibodies (Millipore Sigma; AP132P and AP160P; 1:5000) and Supersignal Femto chemiluminescence reagents (Thermo Fisher Scientific) were used before scanning the membranes with a chemiluminescence scanner (Odyssey). The primary antibodies were purchased from Cell Signaling Technology [anti-GAPDH (2118S, 1:10,000), anti-AKT phosphoS473 (9271, 1:1000), anti-AKT (4691, 1:1000), anti-p44/42 MAPK (ERK1/2) phosphoT202/204 (4695, 2:1000), anti-p44/42 MAPK (ERK1/2) (137F5, 1:1000) anti-EGFR phosphoT1068 (3777, 1:500) and anti-ERBB3 phosphoT1289 (2842, 1:500)], Abcam [anti-active YAP1 (ab205270, 1:1000) and anti-YAP1 phosphoS127 (ab76252, 1:1000)] and Sigma-Aldrich [anti-vinculin (V9131, 1:40,000)].

### RNA sequencing

3′ RNA sequencing was performed by the Biomedicum Functional Genomics Unit at the Helsinki Institute of Life Science and Biocenter, Finland, at the University of Helsinki. 3′ UTR RNAseq ‘Bulkseq’ is based upon the Dropseq method for single-cell sequencing (Macosko et al., 2015). The method involves priming mRNA with an oligo-dT primer that also contains a 12 bp barcode and an 8 bp unique molecular identifier (UMI) sequence, which can be used to remove PCR duplicates during data analysis. Single-stranded cDNA is then converted to double-stranded cDNA using the template-switch effect, and double-stranded cDNA product is then amplified with PCR and another set of primers (SMART PCR primer). Samples were pooled and PCR sequencing pools were made with Nextera i7 primers and the Dropseq P5 primers. Sequencing was performed on the NextSeq High Output 75 cycle flow cell on the NextSeq 500 (Illumina). Quality library metrics were assessed using multiqc tool (Ewels et al., 2016). The fastq files were aligned to the mouse reference genome (GRCm38) using STAR-aligner (v2.7.8a) (Dobin et al., 2013). Samples were demultiplexed and count matrix of UMI counts was created using the ‘CreateDGEMatrix’ command from the suite of tools BRB-seqToolsv1.4 (https://github.com/DeplanckeLab/BRB-seqTools). R2 aligned BAM and R1 fastq files were used as input. The R1 read had a length of 21 nucleotides: 12-nucleotide sample barcode, 8-nucleotide UMI and one extra nucleotide. Raw fastq files, barcode sample information as well as the processed UMI count matrix are available at the Gene Expression Omnibus database (GSE200704). Computational resources for all the data processing were provided by the CSC – IT Center for Science, Finland.

### Bulk RNA-sequencing analysis

Differential expression analysis was performed with DESeq2 package (Love et al., 2014) following the DESeq2 pipeline (https://bioconductor.org/packages/devel/bioc/vignettes/DESeq2/inst/doc/DESeq2.html). In the ‘DESeqDataSetFromMatrix’ function, the design matrix included experiment, treatment and sample replicates. Data were pre-filtered to keep only rows that had at least five read counts. EGF samples were used as a control group. The ‘contrast’ command was used to specify the coefficient of EREG- and NRG1-treated samples relative to that of EGF-treated samples. All the samples in the results table with a ‘BaseMean’ value less than 1 were excluded as well as samples without any ‘padj’ value. Data transformation and visualization were done using variance-stabilizing transformations (Anders and Huber, 2010). Batch removal was performed for experiments and sample replicates using the ‘removeBatchEffect’ function from limma (Ritchie et al., 2015) according to the DESeq2 pipeline. The unsupervised clustering was done using the ‘topVarGenes’ function showing the top 20 genes.

### GSEA

GSEA (Mootha et al., 2003; Subramanian et al., 2005) was performed using the GO_Biological Process dataset as well as the Hallmark (Liberzon et al., 2015), Kyoto Encyclopedia of Genes and Genomes (KEGG), WikiPathways (Martens et al., 2020) and PID (Schaefer et al., 2009) datasets. In addition, the following datasets were analyzed: fetal spheroid (Mustata et al., 2013) – entire upregulated dataset (317 genes), YapTGup {DOX-induced YAP overexpression in the intestinal epithelium; genes with log_2_[fold change (FC)]>2 (306 genes)} (Gregorieff et al., 2015), YapUP (genes overexpressed in YAP WT versus YAP knock-out intestine (log_2_FC>1, 205 genes) (Gregorieff et al., 2015) and intestinal granuloma (Nusse et al., 2018) (entire dataset of upregulated genes in granuloma versus normal intestine, FC>2, 131 genes). The analyses were conducted using the GSEA v4 software (Subramanian et al., 2005). Normalized enrichment scores (NES) and false discovery rates (q-values) are displayed in Fig. 4E.

### scRNA-seq data analysis

scRNA-seq data of murine colonic mesenchyme published by Kinchen et al. (2018) (GSE114374) and Roulis et al. (2020) (GSE142431, samples GSM4227211 and GSM4227215) were acquired from the Gene Expression Omnibus and re-analyzed in R using the Seurat V4 pipeline (Hao et al., 2021). Prefiltering was done to exclude possible doublets as well as cells expressing high levels of mitochondrial or ribosomal RNA. Genes detected in fewer than three cells were initially filtered out as were cells that expressed less than 200 genes. Further prefiltering was done using quality-control metrics. For GSE114374, cells with fewer than 1000 or more than 5000 unique feature counts (‘nFeatures’) and cells with fewer than 2000 or more than 4000 unique molecules detected (‘nCount’) were filtered out. Cells with >5% mitochondrial or >20% ribosomal counts had already been filtered out by the original authors. For GSE142431, cells containing more than 20% mitochondrial or 30% ribosomal counts, cells with fewer than 250 or more than 1500 unique feature counts and cells with fewer than 250 or more than 4000 unique molecules detected were filtered out. The data were normalized (‘SCTransform’), integrated and clustered following the Seurat integration pipeline for SCTransformed data (https://satijalab.org/seurat/articles/integration_introduction.html). Major cell populations were identified by cross-referencing cluster-specific gene expression with established marker genes of different cell types.

### Human CRC cohort

The study included a cohort of 315 patients with CRC operated during 1998-2003 at the Department of Surgery, Helsinki University Hospital. Clinical data were obtained from patient records and survival data were provided by the Finnish Population Registration Centre and Statistics, Finland. The median age of the patients at diagnosis was 67.8 (interquartile range 57.8-67.8) and the median length of disease-specific survival was 5.9 years (range 0.0-18.8). Detailed characteristics of patients are shown in Table S2. The Surgical Ethics Committee of Helsinki University Hospital (Dnro HUS 226/E6/06, extension TMK02 §66 17.4.2013) and the National Supervisory Authority of Welfare and Health (Valvira Dnro 10041/06.01.03.01/2012) approved the study. For tumor tissue microarrays, paraffin blocks of tumor samples from surgical specimens fixed in formalin were collected from the archives of the Department of Pathology, University of Helsinki. Hematoxylin- and Eosin-stained sections were re-evaluated by an experienced pathologist who confirmed the diagnosis. Two 1.0-mm-diameter punches were taken from each tumor block with a semiautomatic tissue microarray instrument (TMA) (Beecher Instruments, Silver Spring, MD, USA). One section was cut from each TMA block, giving two spots from each tumor sample.

### TMA immunohistochemistry

TMA blocks were freshly cut into 4-µm sections, fixed on slides and dried at 37°C for 12 to 24 h. TMA slides were treated in a PreTreatment module (Agilent Dako, CA, USA) with retrieval solution pH 9 (Envision Flex target retrieval solution, DM828, Agilent Dako) for 15 min at 98°C for antigen retrieval. The sections were stained with Autostainer 480S (LabVision, Fremont, CA, USA) using Dako REAL EnVision Detection System, Peroxidase/DAB, Rabbit/Mouse. First, slides were treated with Envision Flex peroxidase-blocking reagent SM801 for 5 min to block endogenous peroxidases. The slides were stained with the anti-NRG1 antibody (ab191139, Abcam; 1:200 diluted in Dako REAL Antibody Diluent S2022) for 60 min at RT. Subsequently all slides underwent a 30-min incubation with peroxidase-conjugated EnVision Flex/HRP (SM802) rabbit/mouse (ENV) reagent. Slides were visualized using DAB chromogen (EnVision Flex DAB, DM827) for 10 min. Mayer's Hematoxylin (S3309, Dako) was used for counterstaining. Brain tissue served as a positive control. The TMA slides were scanned with a digital slide scanner (3DHISTECH Pannoramic 250 FLASH II). Caseviewer software (3DHISTECH) was used to export images and set scale bars.

### Interpretation of the staining

NRG1 immunoreactivity was interpreted independently by two researchers (T.T.L. and J.H.). The researchers were blinded to the clinical outcome of the patients. Negative staining was scored as 0, weakly positive as 1, moderately positive as 2 and strongly positive as 3, separately for cancer/epithelial cells and stromal cells. Of patients with two separate tumor spots, the highest score was considered representative of NRG1 expression. In case of discrepancy between the observers, a consensus score was used. For statistical analysis, samples were grouped into low (negative to low) and high (moderate to high).

### Statistical analysis

The graphs were drawn, and statistical analyses performed using the Prism 9 software. Two-tailed unpaired *t*-test and one-way ANOVA with Tukey's post hoc test were used as statistical tests as detailed in the figure legends. Asterisks denote statistical significance (*P*<0.05). Survival analysis was done by the Kaplan–Meier method and the log-rank test. Disease-specific survival was counted from the day of surgery to the date of death from CRC, or until the end of follow-up. *P*<0.05 was considered significant. All tests were two-sided. The statistical analyses in CRC cohorts were done with SPSS version 27.0 (IBM SPSS Statistics version 27.0 for Mac; SPSS, Chicago, IL, USA, an IBM Company).

## Supplementary Material

10.1242/dmm.049692_sup1 Supplementary information
