# Supplementary material for: Fibroblast-derived EGF ligand neuregulin 1 induces fetal-like reprogramming of the intestinal epithelium without supporting tumorigenic growth
Source: Dis Model Mech. 2023 Apr 3;16(4):dmm049692. doi: 10.1242/dmm.049692 (PMC10110400; doi:10.1242/dmm.049692)
Supplement: Supplementary information [file dmm-16-049692-s1.pdf]

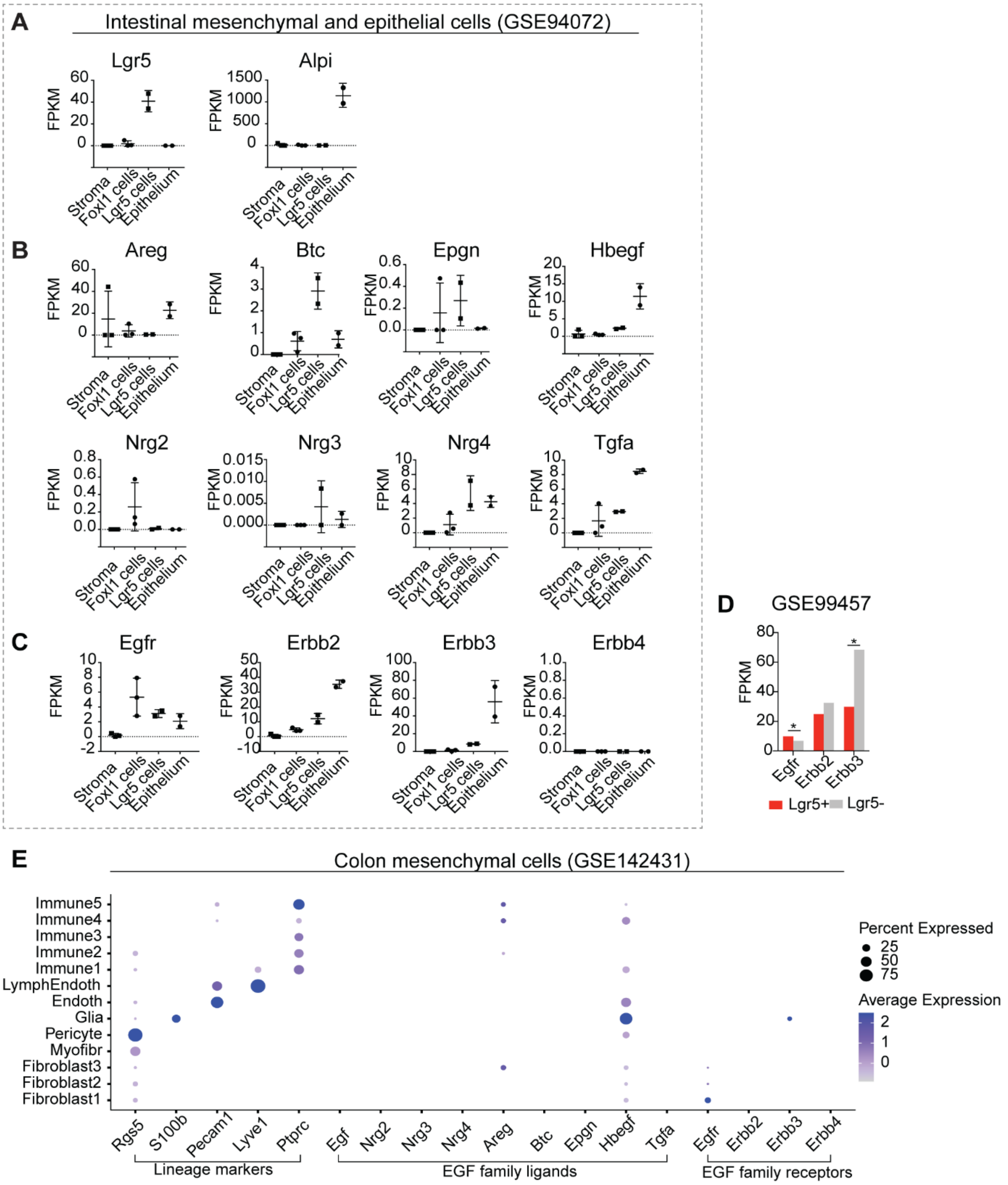

Fig. S1. EGF family ligand expression in mouse GI stroma. (A-C) Comparison of transcript levels of indicated genes in different cell types of mouse small intestine. Non-Foxl1 lineage stromal cells (Stroma), sorted Foxl1-lineage cells representing subepithelial fibroblasts (Foxl1-cells), small intestinal epithelium stem cells expressing Lgr5 (Lgr5-cells) and non-stem epithelial cells (Epithelium) identified by

Shoshkes-Carmel *et al.* (GSE94072). **(A)** Marker genes of stem cells (Lgr5) and mature enterocytes (Alpi). **(B)** Similar analysis of all EGF family ligands. **(C)** Similar analysis of all EGF family receptors. **(D)** Comparison of transcript levels of EGF receptors Egfr, Erbb2, and Erbb3 in Lgr5+ mouse small intestinal epithelial stem cells and Lgr5- non-stem epithelial cells identified from Yan *et al.* (GSE99457). Asterisks indicate statistically significant differences measured by One-Way ANOVA. **(E)** Dot plot of mesenchymal, non-fibroblast cell lineage markers and EGF family ligands and receptors in a mouse colon mesenchyme scRNA-seq dataset (Roulis *et al.*, GSE142431).

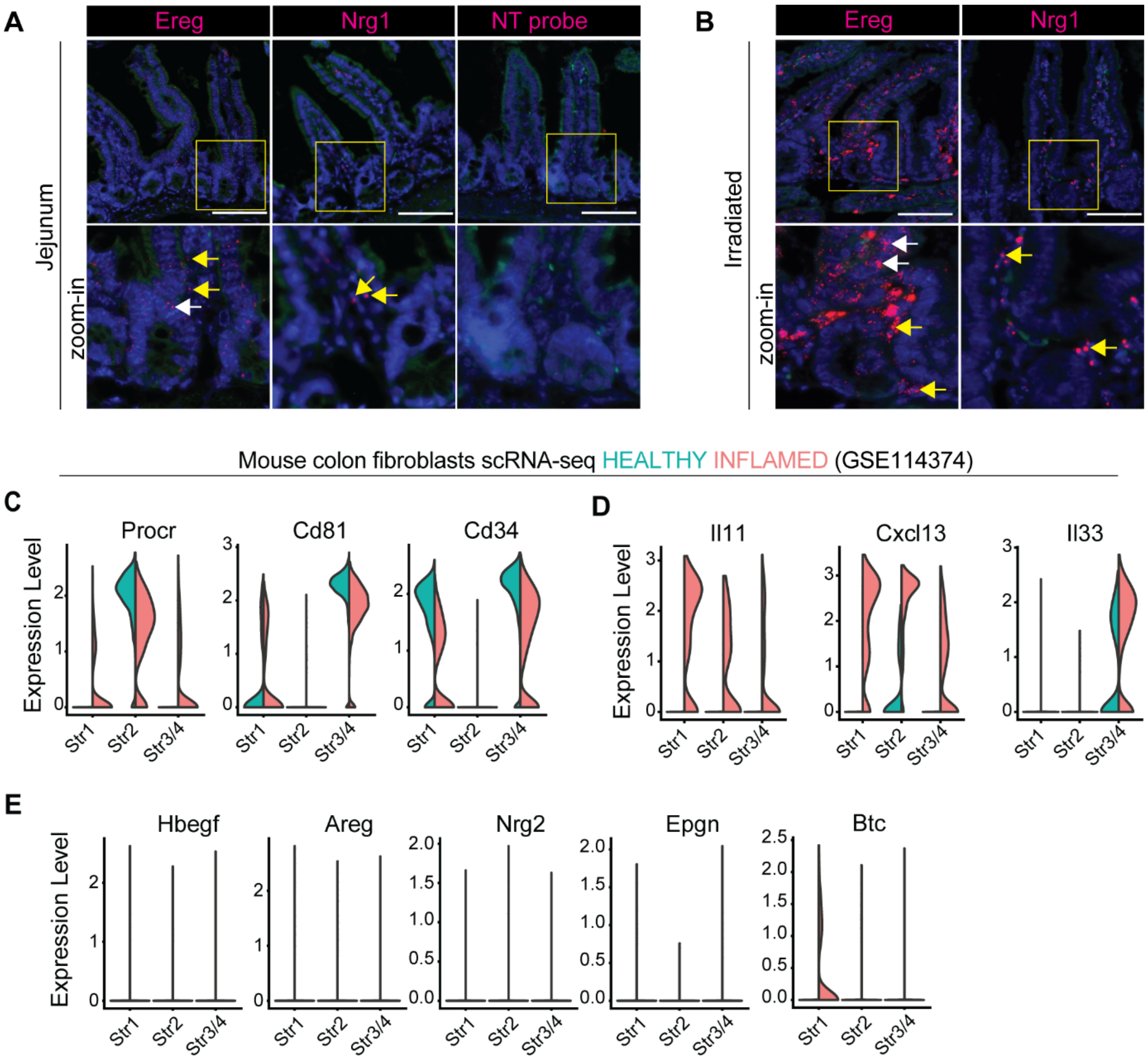

Fig. S2. Fluorescence in situ hybridization and EGF ligand expression in inflammation-activated fibroblasts. (A-B) Fluorescence in situ hybridization of mouse jejunum showing mRNA expression of *Ereg* or *Nrg1*. **(A)** mRNA expression of *Ereg* and *Nrg1* in non-irradiated mouse jejunum. NT probe indicates non-targeting probe control. **(B)** mRNA expression of *Ereg* and *Nrg1* in mouse jejunum four days after 11Gy irradiation. Yellow arrows indicate expression in the stroma, white arrows indicate expression in the epithelium. Green is autofluorescence signal and nuclei (DAPI) in blue. Scale bar 100 µm. (**C-E**) Split violin plots visualizing the expression level of indicated genes in fibroblast clusters isolated from healthy and inflamed mouse colon. Expression level in normal colon fibroblasts is shown in green and expression level in DSS-treated inflamed fibroblasts in red. **(C)** Markers of the fibroblast populations. **(D)** Examples of inflammation-induced genes in fibroblasts. **(E)** EGF family ligand expression in the fibroblast populations.

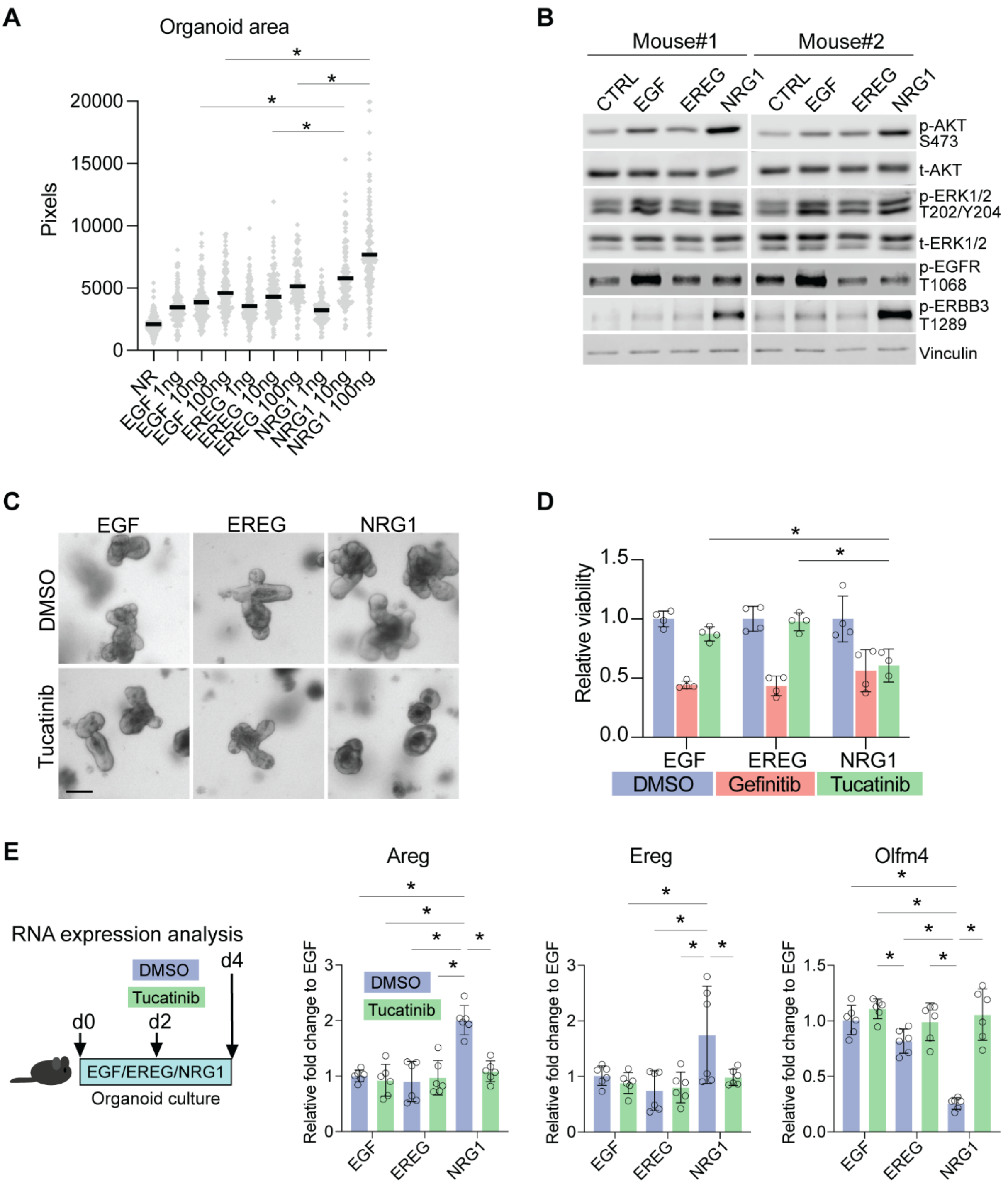

Fig. S3. NRG1 signals via ERBB2/3 and AKT. (A) Area of mouse small intestinal organoids in indicated concentrations of EGF, EREG and NRG1. NR = Noggin + RSPO1 only. n= 107–176 organoids were measured per condition from two independent experiments at day 4 in culture. Black line depicts the mean size of organoid in each group. **(B)** Western blot showing AKT and ERK pathway activation

and EGFR and ERBB3 receptor phosphorylation in freshly isolated small intestinal epithelium incubated for 20 minutes with 100ng/ml EGF, EREG, or NRG1 before lysis. **(C)** Representative figures of mouse small intestinal organoids grown for 3 days in 100 ng/ml EGF, EREG, or NRG1 with and without the ERBB2 inhibitor Tucatinib (2 µM). Scale bar: 100 µm **(D)** Luciferase-based viability assay of mouse small intestinal organoids grown for 3 days with Gefitinib (1 µM) or Tucatinib (2 µM) in the presence of EGF, EREG, or NRG1. A representative of three independent experiments is shown. **(E)** Expression level of indicated genes after 2 days of Tucatinib treatment to small intestinal organoids. Data from two independent experiments. Asterisks denote a statistically significant difference (One-Way ANOVA). Bar graphs depict the mean, error bars represent standard deviation. Asterisks indicate statistical significance ($p < 0.05$, One-Way ANOVA).

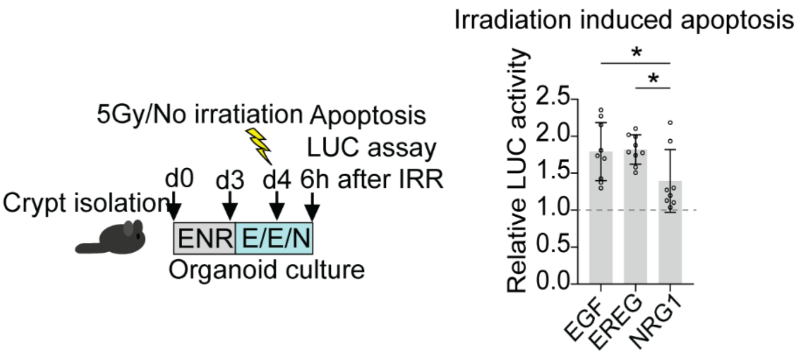

Fig. S4. NRG1-treated small intestinal organoids display reduced irradiation-induced apoptosis. Relative luciferase (LUC) activity representing Caspase 3/7 activity in EGF, EREG and NRG1 treated organoids 6 hours after 5 Gy gamma irradiation. Dashed line represents the no-irradiated control. Three independent experiments with 2-3 datapoints per experiment are shown. Asterisks denote a statistically significant difference (One-Way ANOVA). Bar graphs depict the mean, error bars represent standard deviation.

Disease Models & Mechanisms • Supplementary information

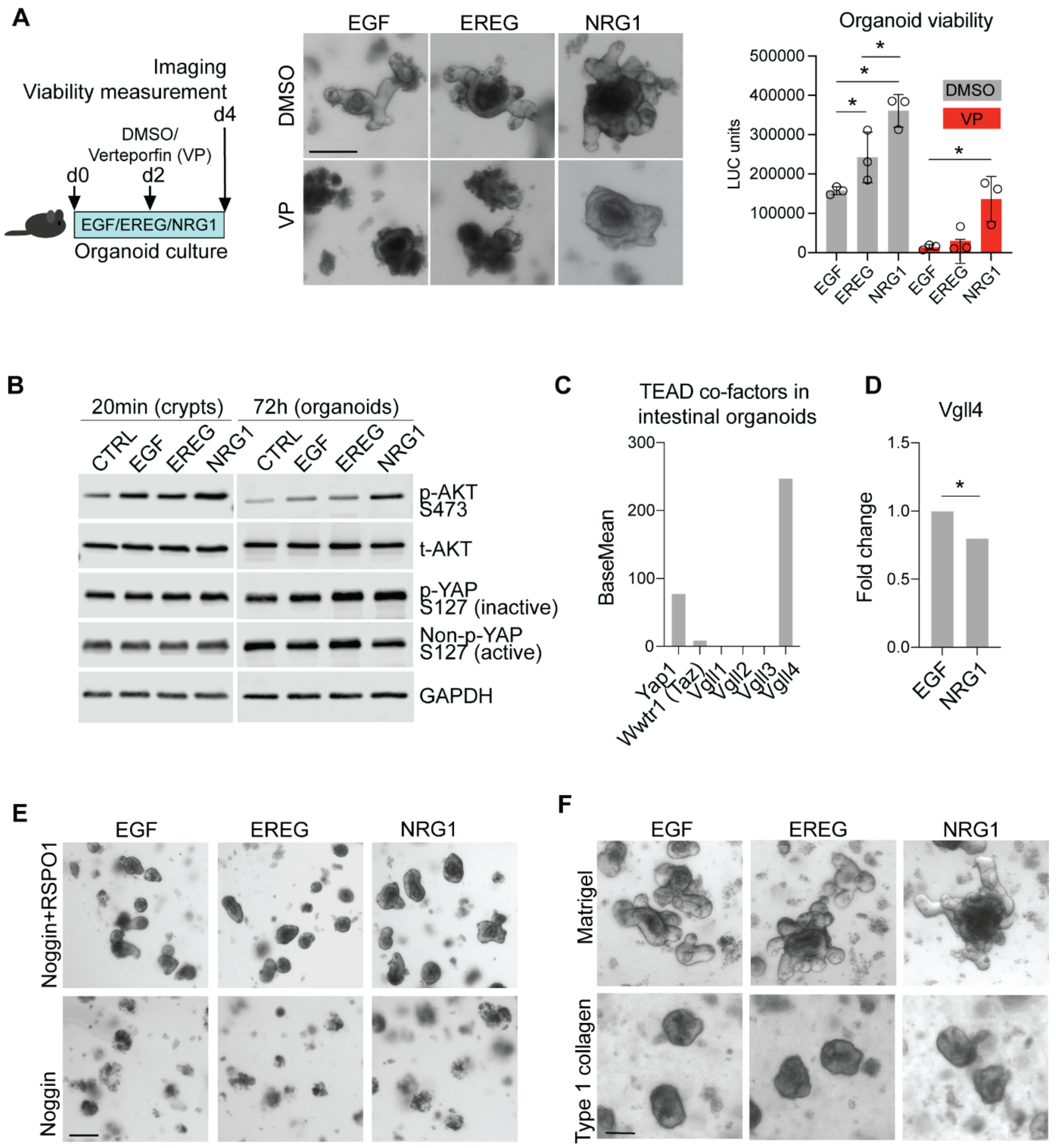

Fig. S5. YAP/TEAD signaling in NRG1-treated small intestinal organoids. (A) Mouse small intestinal organoids were grown in the presence of 100 ng/ml EGF, EREG or NRG1. DMSO or Verteporfin (VP, 1μM) was added at day 2. Representative images from day 4 and a luciferase assay representing organoid viability is shown. Scale bar: 100 μm. The LUC assay is a representative of two independent experiments. Bar graphs depict the mean, error bars represent standard deviation. **(B)** Western blot showing AKT pathway activation (p-AKT S473) and phosphorylated (inactive) and non-phosphorylated (active) YAP S127 in mouse small intestinal epithelium treated with 100ng/ml EGF, EREG, or NRG1. Freshly isolated mouse small intestinal crypts were treated for 20 min (left) and small

intestinal organoids for 72 hours before lysis. **(C)** Expression level estimation (BaseMean) of indicated TEAD co-factors identified in the RNA-seq dataset (this study). **(D)** *Vgll4* expression from RNA sequenced mouse small intestinal organoids treated with EGF or NRG1. Asterisk denotes a statistically significant difference (See Table S1). **(E)** Representative images of mouse small intestinal organoids growing with EGF, EREG, NRG1 with or without RSPO1 at day 4. Scale bar: 200µm. **(F)** Representative images of mouse small intestinal organoids growing in Matrigel or Type 1 collagen with EGF, EREG or NRG1 on day 4. Scale bar: 100 µm.

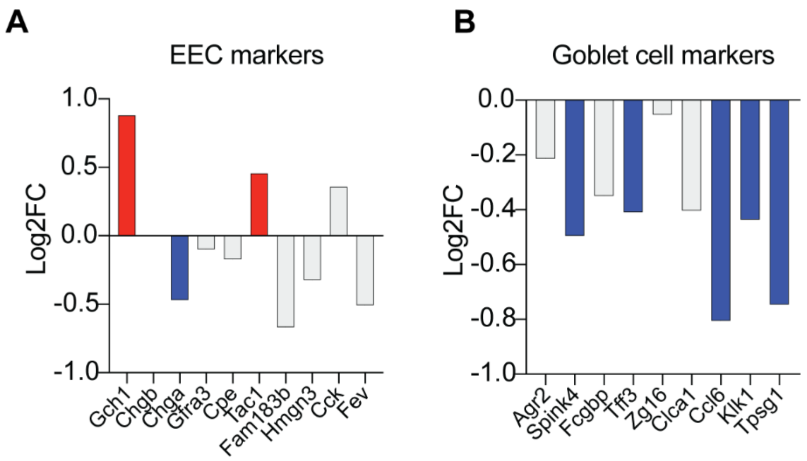

Fig. S6. Expression of enteroendocrine and Goblet cell markers in NRG1-treated small intestinal epithelium. RNA-seq results of NRG1-induced gene expression as compared to EGF-treated small intestinal organoids. Log2 fold change shown for the top 10 marker genes for mouse small intestinal (A) enteroendocrine cells (EECs) and (B) Goblet cells (Haber et al., 2017). The Goblet cell marker gene Muc2 was not identified in the dataset. Statistically significant (p_{adj} < 0.05) changes are highlighted in red (upregulated) and blue (downregulated).

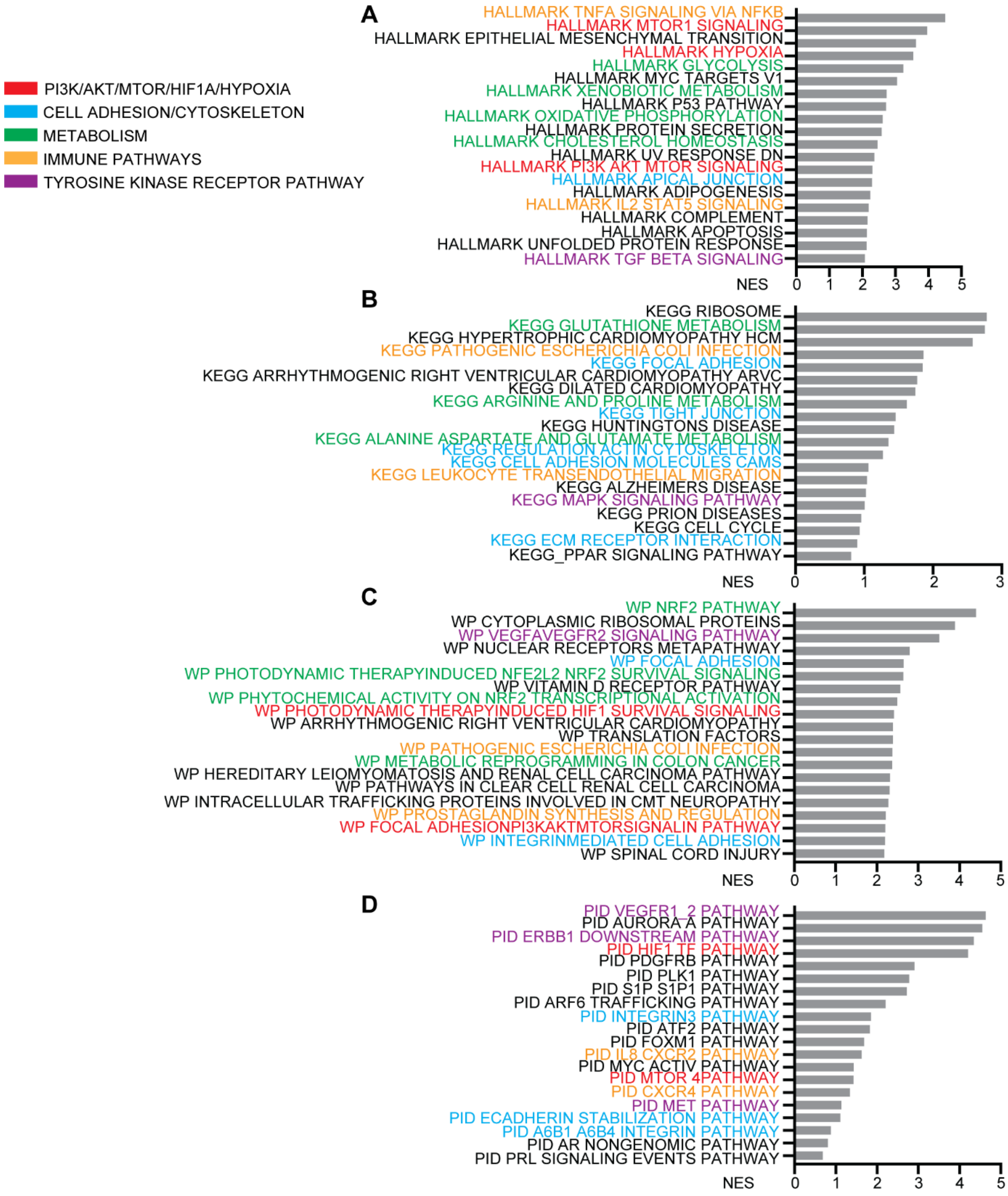

Fig. S7. Unbiased GSEA analysis of NRG1 treatment enriched transcriptome. RNA-seq results of NRG1-induced gene expression as compared to EGF-treated small intestinal organoids. The gene list was ranked by Log2 fold-change. The 20 top pathways enriched in each analysis were ranked by normalized enrichment score (NES). Analysis of Hallmark **(A)**, KEGG **(B)**, WikiPathways **(C)** and PID **(D)** pathways datasets are shown. Pathways related to indicated categories are highlighted with corresponding colors.

Disease Models & Mechanisms • Supplementary information

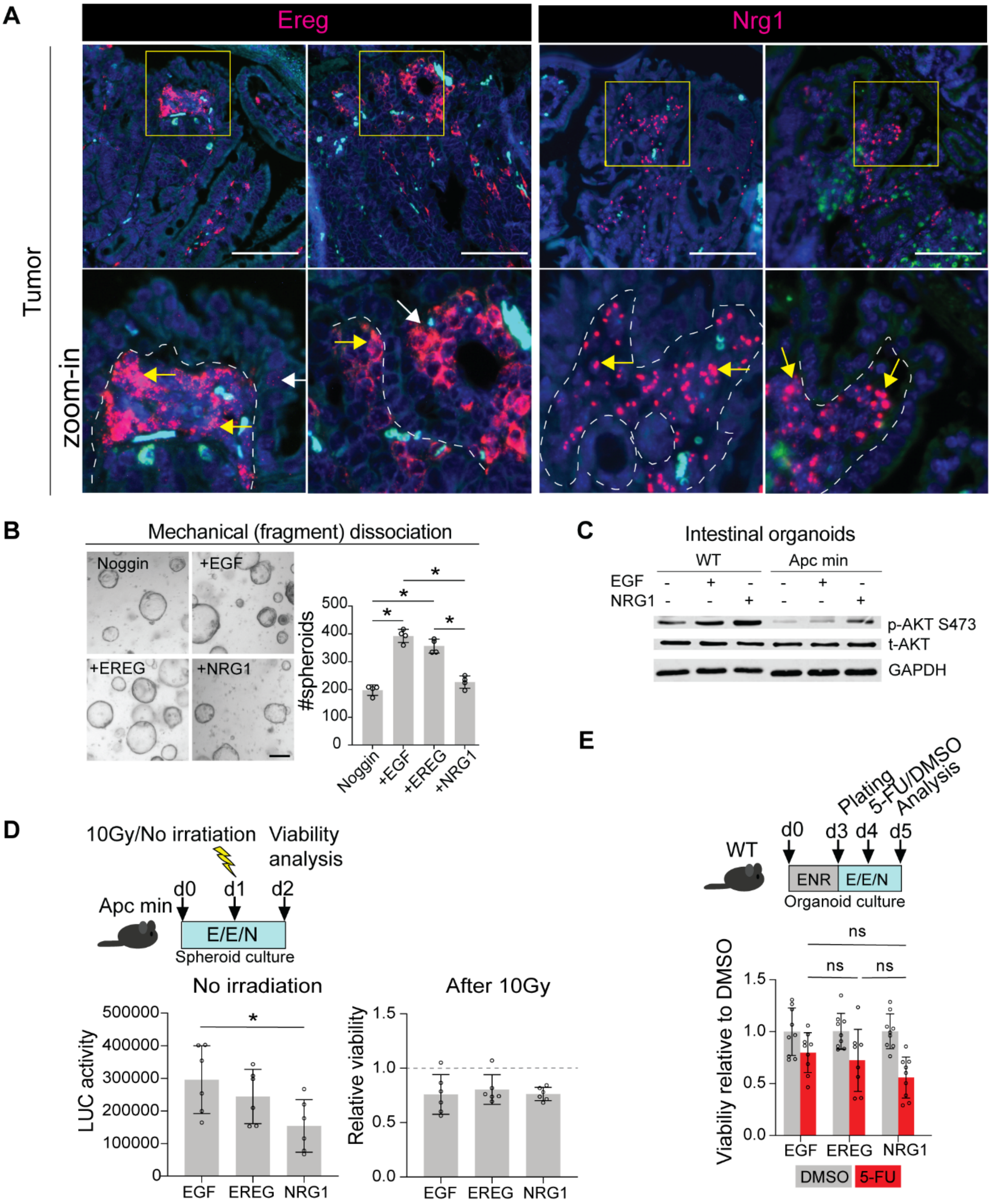

Fig. S8. NRG1 does not protect tumorigenic epithelium under radiation challenge. (A) Fluorescence in situ hybridization of *Ereg* and *Nrg1* in *Apc*^{min} mouse tumors. Dashed line represents the border between stroma and epithelium. Yellow arrows indicate expression in the stroma and white arrows are expression in the epithelium. Green is autofluorescence signal and nuclei is seen in blue. Scale bar:

Disease Models & Mechanisms • Supplementary information

100 µm. **(B)** Culture of pre-tumorigenic *Apc* mutant small intestinal spheroids in media supplemented with EGF, EREG, or NRG1. Experimental design as in Fig. 6A, except that manual dissociation was used to split the spheroids. Representative images and quantification of the number *Apc* mutant spheroids per well in indicated conditions are shown. The experiment shown is a representative of three independent experiments. Scale bar: 200 µm. **(C)** Western blot showing AKT pathway activation (p-AKT S473) in WT and Apc^{min} small intestinal organoids. The organoids were grown for 2 days before collection (WT organoids in ENR and Apc^{min} spheroids in EN) and incubated for 20 min with 100 ng/ml of EGF or NRG1 in basal media. **(D)** Left graph: viability of non-irradiated *Apc* mutant small intestinal spheroids as measured by luciferase (LUC) activity. Right graph: viability relative to non-irradiated small intestinal spheroids (dashed line) after 10Gy gamma irradiation. A combination of two independent experiments with three technical replicates each is shown. **(E)** Viability of WT small intestinal organoids 24 hours after addition of 5-FU or DMSO with indicated treatments. Each datapoint represents one organoid well. A combination of 3 independent experiments with three technical replicates each is shown. Asterisks indicate statistical significance (One-Way ANOVA). Bar graphs depict the mean, error bars represent standard deviation.

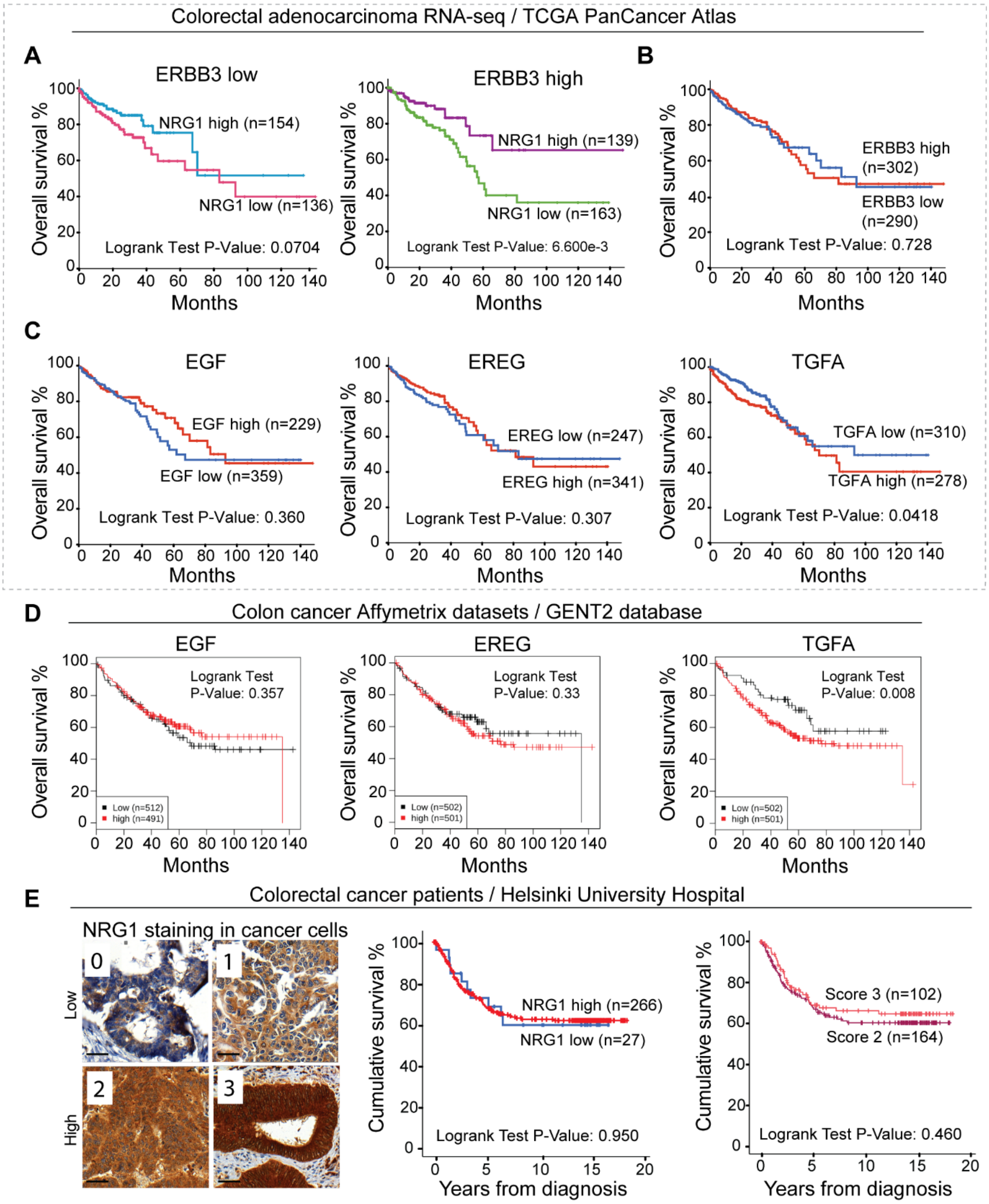

Fig. S9. Correlates with improved survival in CRC. (A) Kaplan-Meier curves depicting the probability of overall survival of CRC patients displaying high (>median) or low (<median) expression of NRG1 mRNA in ERBB3 mRNA low (<median), (left) and ERBB3 mRNA high (>median) patients (right). **(B)** Kaplan-Meier curves depicting the probability of overall survival in CRC patients displaying high (>median)

or low (<median) expression of *ERBB3* mRNA assessed by RNA sequencing. **(C)** Kaplan-Meier curves depicting the probability of overall survival in CRC patients displaying high (>median) or low (<median) expression of *EGF*, *EREG* or *TGFA* mRNA assessed by RNA sequencing. A-C: Data is retrieved from the TCGA PanCancer Atlas. **(D)** Kaplan-Meier curve depicting the probability of overall survival in CRC patients displaying high (>median) or low (<median) expression of *EGF*, *EREG* or *TGFA* mRNA assessed by Affymetrix microarray analysis. Data is retrieved through the GENT2 database. **(E)** Representative figures of NRG1 expression in cancer cells in CRC tissue sections, and Kaplan-Meier curve depicting the probability of disease specific survival in CRC patients displaying high (score 2-3) or low (score 0-1) expression (left graph) and score 2 and 3 separately (right graph). Scale bar: 50µm.

Table S1. RNAseq data from intestinal organoids comparing NRG1 treatment to EGF

Click here to download Table S1

Table S2. Patient characteristics of human CRC cohort

Patient characteristics

	315 n (%)
**Age (median, IQR)**	67.8 (57.8–76.7)
**Gender**	
Male	163 (51.7)
Female	152 (48.3)
**Stage (TNM IV)**	
I	64 (20.3)
II	84 (26.7)
III	111 (35.2)
IV	56 (17.8)
**Grade (WHO)**	
1-2	267 (85.3)
3-4	46 (14.7)
**Side**	
Right	87 (27.6)
Left	228 (72.4)
**Histology**	
Non-mucinous	289 (91.7)
Mucinous	26 (8.3)
**MMR**	
Proficient	266 (85.3)
Deficient	46 (14.7)

MMR=Miss match repair, IQR= Inter quartile range

Table S3. qPCR primer sequences (5′-3′)

Gene	Forward primer (5′-3′)	Reverse primer (5′-3′)
Actb	CTAAGGCCAACCGTGAAAAG	ACCAGAGGCATACAGGGACA
Areg	CTCCACAGGGGACTACGACTA	GGGCTTAATCACCTGTTCAACT
Egf	TGATGGGAAACAATGTCACG	ACCTGCAGGACAGATGCAC
Ereg	ACCGTGATCCCCATCATGC	GGGGATCGTCTTCCATCTG
Lgr5	TAAAGACGACGGCAACAGTG	GCCTTCAGGTCTTCCTCAAA
Mki67	GCTCCTGCCTGTTTGGAAG	TGCTCTTTGACTTCAATTTTGC
Nrg1	TCAGCAAGTTAGGAAACGACAG	ACATGCCAGTGGTGAGGTC
Olfm4	CAGCTGCCTGGTTGCCTCCG	GGCAGGTCCCATGGCTGTCC
Pdgfra	AAGACCTGGGCAAGAGGAAC	GAACCTGTCTCGATGGCACT
Tnf	GATCGGTCCCCAAAGGGATG	TTTGCTACGACGTGGGCTAC

Disease Models & Mechanisms • Supplementary information
